# Random errors in protein synthesis activate an age-dependent program of muscle atrophy in mice

**DOI:** 10.1038/s42003-021-02204-z

**Published:** 2021-06-08

**Authors:** James Moore, Rashid Akbergenov, Martina Nigri, Patricia Isnard-Petit, Amandine Grimm, Petra Seebeck, Lisa Restelli, Stephan Frank, Anne Eckert, Kader Thiam, David P. Wolfer, Dimitri Shcherbakov, Erik C. Böttger

**Affiliations:** 1 Institut für Medizinische Mikrobiologie, Universität Zürich, Zürich, Switzerland; 2 Anatomisches Institut, Universität Zürich, Zürich, Switzerland; 3 Institute of Human Movement, Sciences and Sport, ETH Zürich, Zürich, Switzerland; 4 genOway, Lyon, France; 5 Transfaculty Research Platform Molecular and Cognitive Neuroscience, Universität Basel, Basel, Switzerland; 6 Zurich Integrative Rodent Physiology (ZIRP), Universität Zürich, Zürich, Switzerland; 7 Institut für Pathologie, Universitätsspital Basel, Basel, Switzerland

**Keywords:** Ribosome, Transcriptomics, Protein quality control

## Abstract

Random errors in protein synthesis are prevalent and ubiquitous, yet their effect on organismal health has remained enigmatic for over five decades. Here, we studied whether mice carrying the ribosomal ambiguity (*ram*) mutation Rps2-A226Y, recently shown to increase the inborn error rate of mammalian translation, if at all viable, present any specific, possibly aging-related, phenotype. We introduced Rps2-A226Y using a Cre/loxP strategy. Resulting transgenic mice were mosaic and showed a muscle-related phenotype with reduced grip strength. Analysis of gene expression in skeletal muscle using RNA-Seq revealed transcriptomic changes occurring in an age-dependent manner, involving an interplay of PGC1α, FOXO3, mTOR, and glucocorticoids as key signaling pathways, and finally resulting in activation of a muscle atrophy program. Our results highlight the relevance of translation accuracy, and show how disturbances thereof may contribute to age-related pathologies.

## Abstract (web summary)

By introducing a ribosomal ambiguity mutation into mice, Moore et al. establish an in-vivo model to investigate how age-related diseases are related to decreasing accuracy in protein synthesis. Their findings potentially offer new insights into the pathological changes observed in age-related diseases, such as muscle atrophy

## Introduction

Protein synthesis and folding are intrinsically error-prone, leading to a flux of destabilized and misfolded proteins. The main contributor to this error comes from mRNA decoding by the ribosome which has an average error rate of 10^−4^, making it the limiting factor in the accuracy of gene expression^1^. The idea that aging and age-related diseases may be related to a decrease in protein synthesis accuracy dates back to the early 1960s, culminating in the error catastrophe theory of aging^2,3^. While this theory has been clearly refuted^4^, evidence has been provided, although not undisputed, that aging of mammalian cells in cell culture is accompanied by an increase in protein synthesis error frequency^5,6^, and the corresponding accumulation of protein aggregates^7^. Subsequently, extensive studies in unicellular organisms such as *Escherichia coli* and *Saccharomyces cerevisiae* have provided ample evidence that cellular aging and rejuvenation are associated with asymmetric segregation of protein aggregates^8–10^.

In mammals, translational accuracy has been suggested to positively correlate with maximum possible lifespan amongst different rodent species^11^, illustrating a link between translation accuracy and the seemingly age-accelerating consequences of translation errors. The intrinsic error in protein translation and the dependency on proteome integrity for a long lifespan makes proteostasis a major concern for human health. Constant surveillance by an integrated network of chaperones and protein degradation machineries is required to maintain cellular proteostasis^12,13^. The capacity of the proteostasis network is thought to decline during aging, contributing to the development of age-related diseases^14,15^. In muscle, this takes the form of sarcopenia, the age-associated decline in skeletal muscle function and mass^16,17^. Sarcopenia, which frequently comes with enhanced activity of the ubiquitin-proteasome pathway and elevated levels of polyubiquitylated proteins^18^, is the most frequent cause of disability in the elderly and is associated with frailty and increased mortality^19^.

The inherent errors in mRNA decoding largely result in missense substitutions as per the incorporation of mismatched aa-tRNAs^20^. Due to the physical constraints of the mRNA–tRNA interaction, missense mutations are limited to accommodation of near-cognate aa-tRNAs^21,22^. Mutation A226Y in ribosomal protein Rps2 is the first described ribosomal ambiguity mutation (*ram*) in higher eukaryotes, recently shown to confer misreading and misfolded protein aggregation in human HEK 293 cells^23^. *Ram*s increase the physiological error rate of translation in a stochastic manner by affecting the initial phase of tRNA selection resulting in reduced discrimination against near-cognate tRNAs and in randomly dispersed amino acid misincorporations into the primary sequence of a protein^24,25^.

## Results

### Generation of Rps2-A226Y transgenic mice

To alter translational accuracy in vivo, we chose the ribosomal ambiguity mutation Rps2-A226Y^23^. Concerns that introducing the A226Y mutation into the *Rps2* gene via knock-in might cause embryonic lethality prompted us to produce conditional alleles. Towards this end, we used a strategy consisting of a transcriptional stop cassette flanked by loxP sites inserted in exon 1 and a mutated exon 3 (A226Y). This resulted in heterozygous *Rps2*^*loxP/wt*^ mice (Supplementary Fig. 1). Heterozygous *Rps2*^*loxP/wt*^ mice were crossed with a CMV-Cre transgenic mice line, expressing Cre recombinase ubiquitously under control of the CMV promoter. Despite the use of a Cre deleter line, which has been used in more than 100 models to trigger whole-body Cre-mediated recombination, and the analysis of >200 pups, the resulting heterozygous Rps2-A226Y mutant mice were unexpectedly mosaic for the mutant allele—only mice displaying a partial excision of the targeted allele were identified (Supplementary Fig. 2a). As shown by Southern blot analysis (Supplementary Fig. 1c) the heterozygous *Rps2* A226Y mutant mice (*Rps2*^*A226Y/WT*^) show the presence of three different alleles: *Rps2* wild-type allele, non-excised *Rps2* floxed allele, and excised *Rps2* A226Y mutant allele. To segregate the excised *Rps2* A226Y mutant allele from the non-excised *Rps2* floxed allele we mated three of the partially excised mutant knock-in heterozygous males one generation further with C57BL/6 wild-type females. Germ cells from partially excised *Rps2*^*A226Y/WT*^ mice may bear one of the following *Rps2* alleles: wild type (*Rps2*^*WT*^), A226Y mutant (*Rps2*^*A226Y*^), or floxed (*Rps2*^*loxP*^). While germline transmission of *Rps2*^*WT*^ and *Rps2*^*loxP*^ alleles was observed in 57% and 30% of the progeny, respectively, fully excised mice bearing only the *Rps2*^*A226Y*^ allele have never been detected in the 168 pups analyzed (Supplementary Fig. 2b). Statistically, one-third of the progeny should have inherited the *Rps2* A226Y mutant allele. The failure to obtain fully excised heterozygous animals indicates that the excised *Rps2* A226Y mutant allele cannot be inherited (either the excised allele is not propagated in germ cells or is embryonic lethal) but only the *Rps2* loxP allele, resulting in a model which is genetically mosaic for the *Rps2* A226Y mutant allele.

### Muscle impairment in A226Y mice

**Fig. 1:**
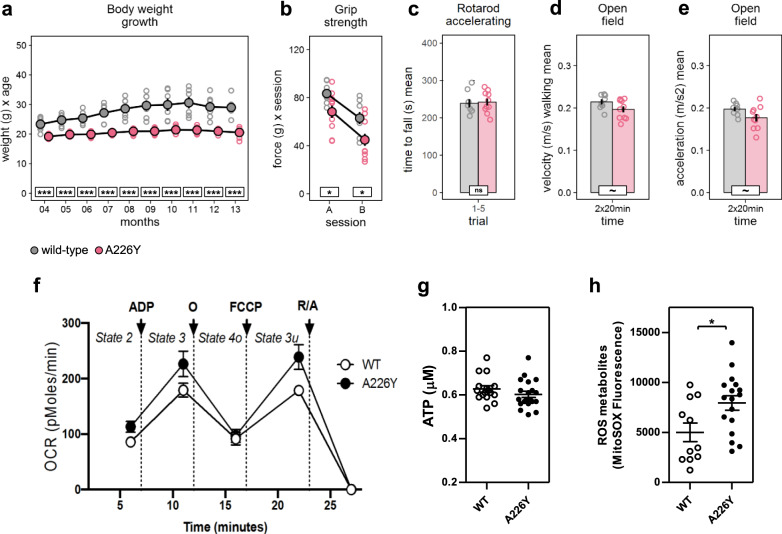
Body weight, behavioral phenotype, and mitochondrial function. Graphs show untransformed mean, SEM and individual data points (gray/white = wild-type mice, red/black = A226Y mice). ^***^p < 0.001, ^**^p < 0.01, ^*^p < 0.05, ~*p* < 0.1, ns *p* ≥ 0.1. **a** Body weight from 4 to 13 months of age (months F1,115 = 87.06, *p* < 0.0001, η² = 0.43; genotype F1,11 = 39.76, *p* < 0.0001, η² = 0.78; genotype × months F1,115 = 5.233, *p* = 0.0240, η² = 0.04, Box-Cox λ 0.0). **b** Forepaw grip strength, assessed during 2 subsequent test sessions of 5 trials (session F1,16 = 29.39, *p* < 0.0001, η² = 0.65; genotype F1,16 = 9.307, *p* = 0.0076, η² = 0.37; genotype × session F1,16 = 0.126 ns). **c** Time to fall off the accelerating rotarod, average of 5 trials (genotype F1,16 = 0.050 ns). **d** Average velocity during walking bouts in the large open-field arena (genotype F1,16 = 3.320, *p* = 0.0872, η² = 0.17). **e** Average acceleration during walking bouts in the large open-field arena (g**e**notype F1,16 = 3.672, *p* = 0.0734, η² = 0.19, Box-Cox λ −0.5). **f** The oxygen consumption rate (OCR) was measured under different respiratory states induced by the sequential injection of ADP (to induce state 3), oligomycin (O, to induce state 4o), FCCP (to induce state 3 uncoupled), and rotenone/antimycin A (R/A, to shut down mitochondrial respiration). Data represent the mean and SEM of *N* = 8 (WT) and *N* = 10 (A226Y) with 2 replicates per animal. Statistical analysis: Two-way ANOVA, multiple comparison between means of WT and A226Y over time (excluding the last time point after R/A injection that is an internal control of the experiment), *p*-value = 0.0063. **g** ATP levels. (13 ≤ *N* ≤ 20). **h** Detection of reactive oxygen species (ROS) production using MitoSOX (11 ≤ *N* ≤ 17). *N* = number of independent mice in each comparison.

No overt mutation-associated abnormalities of spontaneous behavior or general health were noticed in the A226Y mutant mice. However, the A226Y mutant mice presented with a reduced body weight and a flattened growth curve (Fig. 1a). Visual inspection of the animals suggested that the impairment in body weight mainly reflects reduced fat content. Gross inspection during necropsy revealed a visibly smaller amount of adipose tissue in the A226Y mutants as compared to the wild-type control animals. Hematology data did not show any significant difference between A226Y mutants and wild-type controls (Supplementary Fig. 3).

Given the link between proteostasis and neurodegenerative diseases, we assessed a wide spectrum of behavioral phenotypes including changes in learning and memory, anxiety, exploration, and sensorimotor functions. This was done in A226Y mutants and wild-type control mice at 12 months of age to detect early-onset and age-related phenotypes. We tested A226Y mutant mice in a water-maze place navigation task (Supplementary Fig. 4a–c) and in a cued and contextual fear conditioning test (Supplementary Fig. 4d, e) without finding evidence for impaired learning and memory. Examination in a large open field (Supplementary Fig. 4f–h), small open field with home box (Supplementary Fig. 4i), light–dark transition test (Supplementary Fig. 4j), and on the elevated O-maze (Supplementary Fig. 4k) did not reveal any mutation effect on locomotor activity, exploration, or anxiety-related responses. Most likely, the lack of alterations in locomotor activity and cognitive deficits in our A226Y mutants is a result of the limited genetic mosaicism in the brain with less than 5% of A226Y mutant mRNA present (Supplementary Fig. 1d). Attempts to achieve higher and targeted brain expression of *Rps2* A226Y using a neuron-specific Cre-driver line have remained unsuccessful. However, the A226Y mutant mice showed minor impairments in several tests related to motor function (Fig. 1b–e). Forepaw grip strength was reduced in A226Y mutant mice relative to controls (Fig. 1b) during both of the 2 subsequent testing sessions with a mechanical grip force meter. As typically seen in this test, performance was reduced during the second session due to fatigue. We went on to test motor performance on the rotarod. Surprisingly, time to fall off was not significantly affected by genotype (Fig. 1c). However, because performance on the rotarod is negatively correlated with body weight, this does not exclude a mild impairment that may have been masked by the reduced body weight of the A226Y mutant mice. As an additional measure of muscle function, we assessed velocity and acceleration during walking bouts in a large open-field arena and found both velocity and acceleration to be reduced in A226Y mutant mice (Fig. 1d, e). Taken together, these minor changes point to a mild reduction of muscle strength in the A226Y mutants.

### Mitochondrial function in the muscle of A226Y mice

We next asked whether the A226Y mutation affects mitochondrial electron transport chain (ETC) function in muscle. We evaluated the capacity of oxidative phosphorylation in muscle mitochondria of 12 months old A226Y mutant and wild-type control animals using the Seahorse XF24 flux analyzer system. We measured the basal respiration (state 2), the ADP-dependent respiration (state 3), the state 4o (after oligomycin injection), and respiration in the absence of a proton gradient (uncoupled state 3u) (see Fig. 1f). Compared to age-matched wild-type controls, state 2, state 3, and state 3u were in part increased in the A226Y mutants, while total ATP levels were unaffected by the mutation. However, mutants did present an increased production of reactive oxygen species (ROS) metabolites (see Fig. 1g, h).

### Transcriptome analysis

To better understand the subtle muscle-related phenotype of the A226Y mutant animals, we performed whole-genome RNA sequencing. Considering the age-dependency of mammalian sarcopenia, we assessed the transcriptome of skeletal muscle in both mature 9 months and aged 15 months old animals. In skeletal muscle, mutant *Rps2* mRNA was expressed at a level of 20–30% of total *Rps2* mRNA. No significant change in *Rps2* A226Y mRNA expression was observed over time (compare 9 months and 15 months old animals) (Supplementary Fig. 5). On the basis of *Rps2* mRNA expression, we calculate that approximately one-half to one-third of the targeted mutant alleles are excised (see also Supplementary Fig. 1c). These calculations were corroborated by quantitative allele-specific PCRs in muscle tissue which demonstrated that the ratio of mutant *Rps2* A226Y mRNA to wild-type *Rps2* mRNA closely matched the ratio of genomic excised *Rps2* A226Y mutant DNA to genomic wild-type *Rps2* DNA. As muscle fibers are multinucleated cells containing from 50 to several hundred nuclei, and assuming a stochastic distribution of mutant nuclei we conclude that each muscle fiber is affected by the mutation with approximately one-half to one-third of nuclei carrying the excised mutant allele.

In wild-type mice, the transcriptomes of the 9 and 15 months old animals were well separated and gene expression differences revealed changes typical of aging^26^, including a decrease in enrichment terms associated with translation, mRNA processing, ubiquitin-dependent proteasomal degradation, mitochondrial function, and oxidative phosphorylation (Supplementary Fig. 6). To further control for aging-related transcriptomic changes in the control cohort, we projected the muscle transcriptome of the 9 and 15 months old wild-type animals on a previous between-group analysis (BGA) of 3 and 19 months old wild-type animals of the same C57BL/6 genetic background from another study^27^. In an age-dependent manner, the transcriptome of the 9 and 15 months old mice fell within the age boundaries defined by the 3 and 19 months old animals (Supplementary Fig. 6).

### Age-dependent changes in the muscle of A226Y mice

**Fig. 2:**
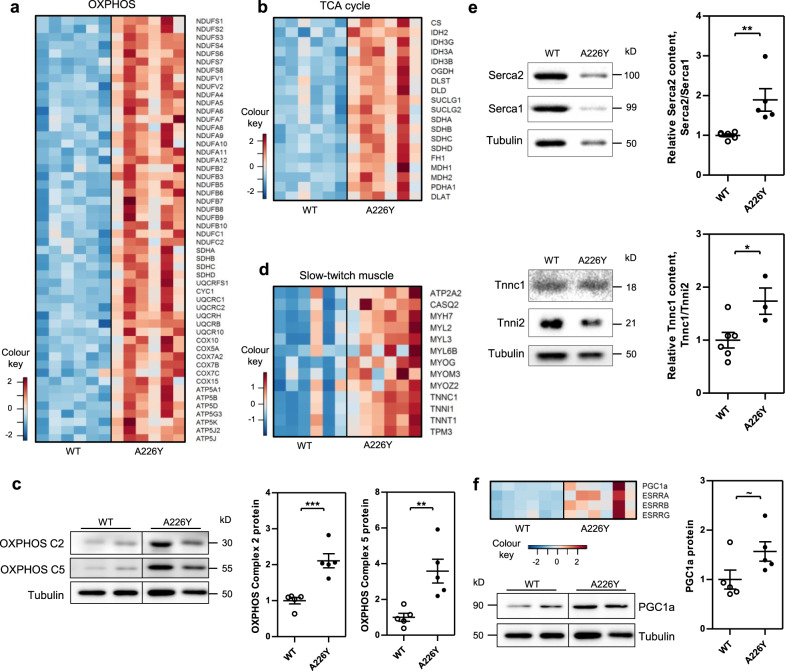
Impact of mistranslation in the muscle of Rps2 A226Y mutant mice at 9 months of age. Graphs show mean, SEM, and individual data points. ^***^p < 0.001, ^**^p < 0.01, ^*^p < 0.05, ~*p* < 0.1. **a** Heatmap showing the significantly regulated genes (FDR < 0.05) in the KEGG pathway ‘Oxidative Phosphorylation (mmu00190)’. **b** Heatmap showing the significantly regulated genes (FDR < 0.05) in the KEGG pathway ‘Citrate cycle (TCA cycle) (mmu00020)’. **c** Western blot and densitometry of OXPHOS Complex 2 (SDHB) and OXPHOS Complex 5 (ATP5A) proteins as indicators of mitochondrial content in 9 months WT and A226Y (*N* = 5). Tubulin as loading control. **d** Heatmap showing the significantly regulated genes (*p* < 0.05) associated with slow-twitch muscle fibers. **e** Western blots of Serca1 and Serca2 levels, and Tnnc1 and Tnni2 levels, in WT and A226Y mouse skeletal muscle. Tubulin as loading control. Graphs show the densitometry of Serca2/Serca1 and Tnnc1/Tnni2 ratios. All values were first normalized to tubulin as loading control (3 ≤ *N* ≤ 6). **f** Heatmap showing expression of key regulators of mitochondrial biogenesis: PGC1a, ESRRA, ESRRB, and ESRRG. Western blot plus densitometry of PGC1a protein level in 9 months WT and A226Y (*N* = 5). Tubulin as loading control. *N* = number of independent mice in each comparison.

The muscle transcriptome of the Rps2-A226Y mice revealed significant mutation-dependent changes in an age-related manner. At 9 months of age, compared to the age-matched wild-type, we noticed a burst of gene expression reflecting increased mitochondrial activity (e.g., mitochondrial organization, mitochondrial translation, oxidative phosphorylation, ETC, TCA, ATP synthesis) as well as enrichment of metabolic pathways (Fig. 2a, b and Table S1). The transcriptome data were further supported by western blot analysis and demonstrated an increased expression of OXPHOS proteins in A226Y mutant mice (Fig. 2c). Along with this metabolic shift came significantly increased expression of genes associated with oxidative slow-twitch muscle fibers, e.g., *Tnnt1, Tnni1, Tnnc1, Tpm3, Myh7, Myl2, Myl3*, and *Atp2a2* (Fig. 2d). Relative increases in the expression of slow-twitch muscle proteins, Serca2 and Tnnc1, to fast-twitch muscle proteins Serca1 and Tnni2, respectively, were corroborated by western blot (Fig. 2e). Importantly, we found increased expression of the peroxisome proliferator receptor γ co-activator 1α (*PGC1α*) and of estrogen-related receptors (ERRs) (Fig. 2f). PGC1α promotes fiber-type switching from glycolytic type II fast-twitch fibers to more oxidative type I slow-twitch fibers, and together with ERRs is a prime regulator of mitochondrial function and oxidative metabolism^28–30^. In all, 18/25 mitochondrial genes, identified in a previous study of C2C12 muscle cells as most highly expressed upon transfection with *PGC1α*^31^, showed significantly increased expression in the A226Y mutant mice (Supplementary Fig. 7). To expand our analysis we used the software CEMiTool^32^ to identify modules of co-regulated genes, as well as putative hubs of gene co-regulation. At 9 months of age, the module with the highest correlation to the A226Y mutant group had top enrichments for mitochondria (GO:0005739, *p* = 2.4 × 10^−9^) and muscle filament sliding (GO:0030049, *p* = 3.6 × 10^−8^). The top hub gene of this module was *PERM1* (PGC1α and ERR induced Regulator, Muscle 1). *PERM1* regulates the expression of selective *PGC1α* and *ERR* target genes with roles in muscle mitochondrial biogenesis, contractile function, energy transfer, and oxidative capacity ^33,34^.

**Fig. 3:**
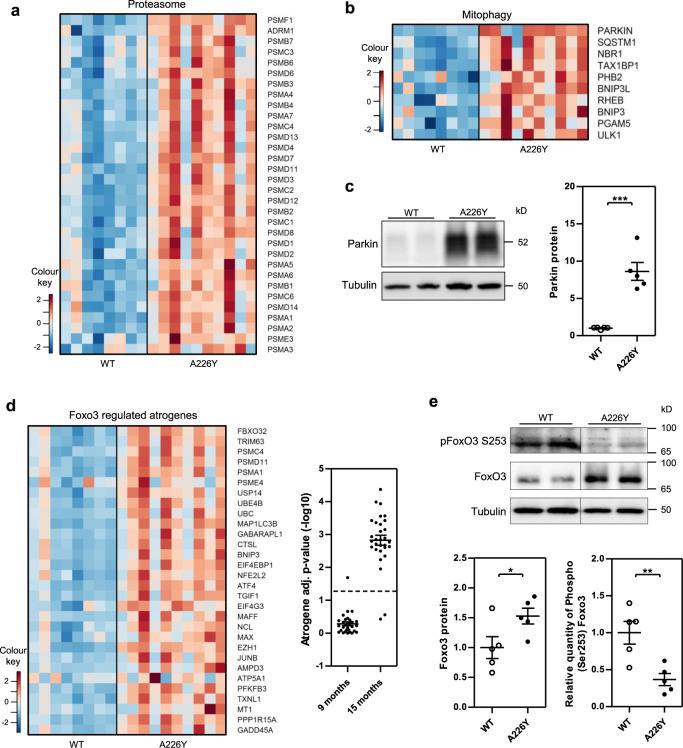
Protein degradation pathways in the muscle of Rps2 A226Y mutant mice at 15 months of age. Graphs show mean, SEM and individual data points. ^***^p < 0.001, ^**^p < 0.01, ^*^p < 0.05. **a** Heatmap representing the significantly upregulated genes (*p* < 0.05) in the KEGG pathway ‘Proteasome (mmu03050)’. **b** Heatmap representing the significantly upregulated genes (FDR < 0.05) involved in priming mitochondria for mitophagy^35^. **c** Western blot and densitometry of total Parkin content in A226Y vs WT (*N* = 5). Tubulin as loading control. **d** Heatmap of Foxo3-regulated atrogenes^39^, and a plot of –log10 FDR adjusted *p*-values for each Foxo3-regulated atrogene (*N* = 30) (A226Y vs WT) in 9 and 15 months mouse muscle. The dashed line indicates *p* = 0.05. **e** Western blot and densitometry of total and specific phosphorylated Foxo3 protein (*N* = 5). Tubulin as loading control. Relative quantity of phosphorylated protein calculated as phosphorylated/total. *N* = number of independent mice in each comparison.

Rps2-A226Y mice at 15 months presented a very different transcriptome profile. Comparison to age-matched wild-type control animals revealed increased expression of terms associated with ubiquitin-dependent proteasomal degradation and RNA processing (Fig. 3a and Table S2). This came along with an enrichment of terms implicated in various proteostatic responses together with a depletion of functional terms representing metabolic pathways, in particular reflecting decreased amino acid metabolism (Fig. 3a and Table S2). Macroautophagy and mitophagy appeared increased in the 15 months A226Y mice transcriptome. By gene expression alone it is difficult to untangle the specific regulation of mitophagy from macroautophagy, however, focusing on those genes which prime mitochondria for mitophagy^35^, we found many of them to be highly expressed in A226Y mutants (Fig. 3b), and further inspection of Parkin protein content by western blot (Fig. 3c) revealed a stark increase in A226Y mice.

Atrophy-related genes (known as atrogenes) are a group of genes that are commonly up- or downregulated in atrophying muscles during different catabolic conditions^36,37^. A large set of these genes is controlled by the Forkhead-Box (FOX) O family of transcription factors, in particular FOXO3^37–39^. We found a significant enrichment for FOXO3-dependent atrogenes in the 15 months A226Y mutants (Fig. 3d). The activity of the FOXO3 transcription factor is controlled by phosphorylation, with exclusion of phosphorylated FOXO proteins from the nucleus and inhibition of their transcriptional function^38^. Assessment of total and phosphorylated FOXO3 by immunoblot and densitometric analysis revealed an increase in the total amount of FOXO3 accompanied by reduced levels of phospho-FOXO3 in A226Y mice (Fig. 3e).

**Fig. 4:**
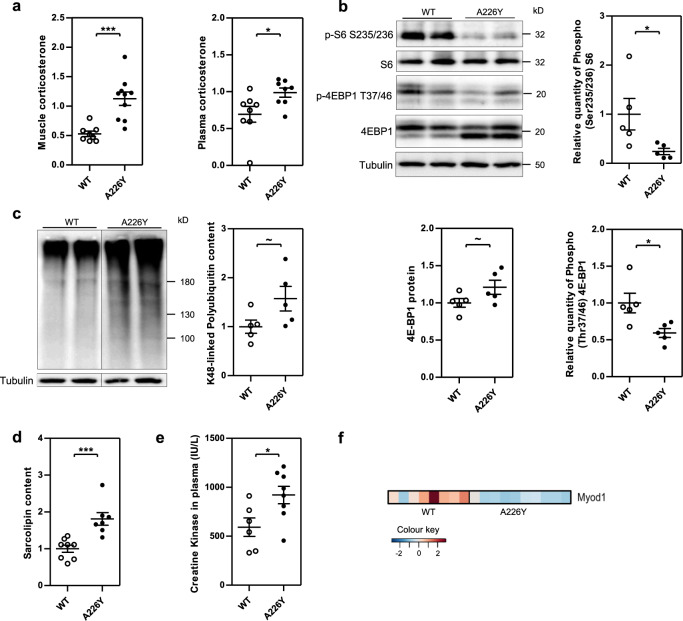
Impact of Rps2 A226Y mutation in mice at 15 months of age. Graphs show mean, SEM and individual data points. ^***^p < 0.001, ^**^p < 0.01, ^*^p < 0.05, ~*p* < 0.1. **a** Corticosterone level in muscle and plasma in WT and A226Y mice (8 ≤ *N* ≤ 10). **b** Western blot and densitometry of total and specific phosphorylated S6 and 4E-BP1 protein in WT and A226Y muscle (*N* = 5). Tubulin as loading control. Relative quantity of phosphorylated protein calculated as phosphorylated/total. **c** Western blot and densitometry of polyubiquitylated proteins in WT and A226Y muscle (*N* = 5). Tubulin as loading control. **d** Total sarcolipin content in the muscle of WT and A226Y mice as measured by ELISA (*N* ≤ 7). **e** Creatine kinase levels in plasma (IU/l) (6 ≤ *N* ≤ 8). **f** Heatmap showing Myod1 mRNA expression in the muscle of WT and A226Y mice (adjusted *p*-value = 0.000813). *N* = number of independent mice in each comparison.

Many pathological conditions characterized by muscle atrophy, e.g., sepsis, cachexia, and starvation are associated with an increase in glucocorticoid levels^40,41^. Transcriptome analysis revealed a significant increase in the expression of established glucocorticoid receptor (GR) target genes in A226Y mutant mice of 15 months of age (Supplementary Fig. 8), including the potent muscle growth inhibitor myostatin (*MSTN*), which can itself cause muscle wasting when in excess^42,43^. This was associated with significantly increased levels of corticosterone both in plasma and muscle (Fig. 4a). Increased GR activity was accompanied by decreased phosphorylation of the mTOR downstream targets 4E-BP1 and S6 (Fig. 4b). In addition to the transcriptomic enrichment for terms implicated in catabolic processes and proteostatic responses, we found increased levels of polyubiquitylated proteins in muscle from 15 months A226Y mutants (Fig. 4c). As a further marker of muscle dysfunction, we assessed the levels of sarcolipin in muscle^44^. Compared to the control animals, the A226Y mutants showed significantly increased levels of sarcolipin (Fig. 4d), combined with decreased expression of *MyoD* (Fig. 4f), a master regulatory gene of skeletal muscle differentiation, development, and regeneration^45^. The overall finding of disturbances in muscle physiology is further supported by increased plasma levels of creatine kinase in the A226Y mutant mice compared to the wild-type controls (Fig. 4e).

To assess whether the various alterations observed in the muscle of the A226Y mutants manifest at the histopathological level, we performed extensive studies of hindlimb skeletal muscle (see Supplementary Fig. 9). However, no signs of fiber atrophy or fiber-type switching were observed, and diameters of type I and II fibers were within normal range in soleus and gastrocnemius muscles (between 25 and 30 μm in both genotypes). Likewise, correlates of muscle dystrophy (nuclear internalization, fiber splitting, increased peri- and endomysial connective tissue, fiber degeneration) were lacking, and storage of glycogen and neutral lipids was similar to wild-type controls. In addition, no autophagy flux impairment was evident on the histological level, as reflected by stains for acidic phosphatase comparable to wild-type animals. Finally, no inflammatory infiltrates were noted.

## Discussion

The limitations of genetic mosaicism in the A226Y model notwithstanding, our findings point to a prominent age-dependent response to error-prone protein synthesis in skeletal muscle. Presumably, this reflects the dynamic expression of main players involved in muscle metabolism: PGC1α, FOXO3, mTOR, and GR. In 9 months old animals we observe increased mitochondrial metabolism together with increased expression of genes and proteins associated with slow-twitch oxidative type 1 muscle fibers. Fiber-type switching together with enhanced myogenin expression is a prominent transcriptome characteristic in mouse models of Duchenne muscular dystrophy^46^. The increased oxidative metabolism in the 9 months old A226Y mice is most likely driven by increased *PGC1α* and *ERR* gene expression. PGC1α promotes the switch to oxidative muscle fibers and together with ERRs is a prime regulator of mitochondrial function and metabolism^28,29^. A general increase in mitochondria-related metabolism is indicated by the increased expression of gene pathways associated with amino acid and lipid metabolism (Table S1). In addition, previous studies have highlighted the role of PGC1α as a dominant antagonist of FOXO3. In various rodent models, muscle atrophy comes along with inhibition of *PGC1α* mRNA expression, whereas *PGC1α* overexpression protects mice from muscle atrophy by suppressing FOXO3 action and atrogene-specific transcription^47^.

In aged 15 months old A226Y mice we observe a degenerative catabolic response, characterized by a transcriptome profile which is surprisingly similar to that of sarcopenic animals^48^. It is, however, still a matter of debate whether the gene expression patterns in sarcopenia are different from those observed in other settings of skeletal muscle loss, e.g., skeletal muscle atrophy caused by denervation, immobilization, or cachexia^49^. Despite clear genotype-specific differences in transcriptional profiles, we do not find overt histopathological signs of dystrophic muscle in the A226Y mutants. However, the results from the sensorimotor tests point to a compromised muscle function, supported also by increased levels of sarcolipin in muscle and creatine kinase in plasma (Fig. 4d, e). In as much as decrements in muscle strength precede changes in histopathology in the context of age-associated muscle loss in humans and mice^50,51^, normal skeletal muscle histopathology is not at all uncommon in patients presenting with subclinical or mild myopathy symptoms ^52^.

Muscle of aged 15 months A226Y mutant mice showed increased levels of both corticosterone and transcripts of GR target genes (see Fig. 4 and Supplementary Fig. 8). Glucocorticoids have been shown to stimulate FOXO3 expression^38,53^ and we hypothesize that it is the interplay between decreased PGC1α expression and increased corticosterone levels in aged 15 months old A226Y mutant mice, which allows FOXO3 to take over, while at the same time resulting in decreased mTOR signaling. Together with elevated levels of ubiquitin-dependent proteasomal degradation, the FOXO3-regulated atrophy program in the 15 months old mutants came along with significantly increased expression of Parkin, a key regulator of mitophagy, pointing to the recently proposed role of mitochondria in cytosolic proteostasis^23,54^. In contrast to the 15 months old animals, which show a prominent induction of FOXO3-regulated atrogenes (Fig. 3d), none of the FOXO3-regulated atrogenes was found to be increased at the transcriptome level in the 9 months animals, with the exception of *ATP5a1* which is part of the ETC (complex V) and regulated by PGC1α. Nor did we find increased levels of polyubiquitylated proteins in the muscle of 9 months A226Y mutants (Supplementary Fig. 10a). Conversely, and as compared to the 9 months A226Y mutant animals, little, if any, increases in the expression of PGC1α gene targets were observed in the 15 months mutants (Supplementary Fig. 7). Moreover, in direct contrast to 9 months, expression of pathways associated with amino acid metabolism was decreased at 15 months (Table S2), indicating reduced metabolic activity. Most notably, we found that Akt, a major upstream regulator of mTOR and FOXO3^55^, as well as GSK3B^56^, are not involved in the muscle pathogenesis of the 15 months A226Y mutants. Assessment of the phosphorylation status of these regulators by immunoblotting and densitometric analysis indicated similar levels of phosphorylated Akt and GSK3B in the 15 months A226Y mutants as compared to the age-matched controls (Supplementary Fig. 11). mTORC1 controls protein synthesis via signaling to S6 and 4E-BP1 and is essential for the maintenance of muscle mass and function^57^. In contrast, glucocorticoid levels are increased in many pathological conditions associated with muscle loss^58^. More recently, a mutually exclusive crosstalk between the catabolic processes provoked by glucocorticoids and the anabolic mTOR pathway has been described^53^, which is what we observe in our mutants. Muscle of 15 months A226Y mutant animals show increased GR catabolic responses combined with decreased signaling of the mTOR pathway, as indicated by decreased phosphorylation of the mTOR downstream targets S6 and 4E-BP1.

In summary, by means of successful introduction of the Rps2 A226Y ribosomal ambiguity mutation into the genome of mice, we have established an in vivo experimental model to investigate the long-standing idea that aging and age-related diseases may be related to the accuracy of protein synthesis. While the present model is limited by genetic mosaicism our findings based on combining proteome-wide mistranslation with system approaches may offer new insights into the pathological changes observed in aging and age-related diseases.

## Methods

### Generation of knock-in *Rps2* transgenic mice

The *Rps2* A226Y mutant mice were generated by knock-in using a targeting vector displaying a transcriptional STOP cassette flanked by *lox*P sites inserted in exon 1 in the 5ʹ UTR upstream of the ATG start codon and a mutated exon 3 (A226Y; GCC-TAC) (Supplementary Fig. 1). The gene-targeting vector was constructed from genomic 129 Sv mouse strain DNA. Upon Cre induction, the *lox*P-STOP-*lox*P cassette will be excised resulting in the expression of the mutant gene. Linearized targeting vectors were transfected into 129 Sv ES cells and positive selection was started 48 h after electroporation, by addition of G418. Resistant clones were isolated, amplified, duplicated, and genotyped by both PCR and Southern blot analysis.

The following primer pair was designed to specifically amplify the targeted locus. 34908sa: 5ʹ-GGTTGTGGCTGACAGCTAGAGAACCA-3ʹ and 0069-Neo-16118sa: 5ʹ-GGGGTGGGATTAGATAAATGCCTGC-3ʹ. Gene targeting was confirmed by Southern blot analysis using internal and external probes on both 5ʹ and 3ʹ ends. PCR and Southern blot genotyping led to the identification of 5 targeted clones. Recombined ES cell clones were microinjected into C57BL/6 blastocysts and gave rise to male chimeras. Breeding with wild-type C57BL/6 mice was conducted to produce the *Rps2*^*loxP/WT*^ line. These mice were backcrossed to C57BL/6 for 7 generations. The heterozygous *Rps2*^*loxP/WT*^ line was crossed with C57BL/6 CRE deleter mice (CMV-Cre) to produce the heterozygous *Rps2* A226Y mutant line for the expression of the mutant *Rps2* A226Y allele. These heterozygous *Rps2* mutants (*Rps2*^*A226Y /WT*^) are referred to as *Rps2* A226Y mutant mice. For each line, heterozygous mice were genotyped by PCR, Southern blot, and sequencing.

*Rps2*^*loxP/WT*^ and *Rps2*^A226*/WT*^ mice were first identified by PCR using a combination of two PCR designs. One PCR specifically amplifies the *Rps2*^*loxP*^ allele using primer 49230sa-EBO6: 5ʹ-GGTTGTGGCTGACAGCTAGAGAACCA-3ʹ and primer 0069-Neo-16118sa: 5ʹ-GGGGTGGGATTAGATAAATGCCTGC-3ʹ. These primers result in the amplification of a specific 2.3 kb fragment for the *Rps2*^*loxP*^ allele, while wild-type *Rps2* does not result in an amplicon. A second PCR specifically amplifies the mutant *Rps2* A226Y allele using primer 49237cof-EBO6: 5ʹ-GGACAGACACAGTTTTGGCAGGACC-3ʹ and primer 49238cof-EBO6: 5ʹ-CTTGTCTTCAGCTTTACCTCCACGAGC-3ʹ. These primers result in the amplification of a 343 bp fragment for the *Rps2*^*A226Y*^ mutant allele. PCR-positive heterozygous *Rps2*^*loxP/WT*^ mice and heterozygous *Rps2*^*A226Y/WT*^ mice were confirmed by Southern blot analysis using the 5ʹ external probe (amplified using the following primers: 5ʹ-ATGGGAGTGGATGCTCAGAGCTACCTT-3ʹ / 5ʹ-CAGCCCTCCTGACTGCAAGTTCCT-3ʹ). Upon digestion with AvrII, a 7.1 kb fragment is expected for the *Rps2*^*WT*^ allele, a 9.0 kb fragment is expected for the *Rps2*^*loxP*^ allele, and a 5.9 kb fragment is expected for the *Rps2*^*A226Y*^ allele (Supplementary Fig. 1). The presence of the mutation in the *Rps2*^A226/*/WT*^ mice was verified by sequencing of a 664 bp product, using primers 49249seq-EBO6: 5ʹ-TCACCTAAGTCTTCTCATAGGCTTTCGTCG-3ʹ and 49250seq-EBO6: 5ʹ-GTAAGAGGGTTGGTGGCTTCATTAGAGTCC-3ʹ.

Breeding of the *Rps2*^*loxP/WT*^ animals with the CMV-Cre line resulted in 345 pups. Analysis of the 345 pups deriving from this breeding revealed 28 animals with Cre-mediated recombination of the A226Y mutation. This recombination was partial, suggesting that the mutant gene/expression did not occur in all cells. Based on standard Mendelian ratio, 86 (25%) out of 345 pups analyzed should have been *Rps2*^A226Y/Cre^ and among them 90–100% should have displayed a full Cre-mediated recombination. To segregate the excised *Rps2*^*A226Y*^ mutant allele from the floxed non-excised *Rps2*^*loxP*^ allele, we mated three of the partially excised heterozygous males one generation further with C57BL/6 wild-type females. Analysis of the resulting 168 pups did not reveal fully excised mice bearing only the *Rps2*^*A226Y*^ allele (Supplementary Fig. 2). The partially excised animals, referred to as A226Y mutant mice (*Rps2*^*A226Y/WT*^) were further investigated by Southern blot analysis and the presence of both the *Rps2 loxP* and the *Rps2 A226Y* alleles was confirmed, in addition to the wild-type allele (Supplementary Fig. 1c). Despite the large number of mice analyzed (*N* = 513), we did not identify animals displaying a full excision. The level of excision was further estimated by Taqman-PCR using genomic DNA as template and a combination of primers and probes which differentiate between wild-type *Rps2*, mutant non-excised *Rps2* (loxP), and mutant excised *Rps2* alleles.

Mice tissue sections were frozen until RNA extraction was performed. RNA was extracted using Trizol reagent (Thermo Fisher) according to the manufacturer’s instructions. The purified RNA was DNase-treated and cDNA synthesis was performed using SuperScript II cDNA synthesis kit (Life technologies) using random hexamers and 40 ng RNA in 20 µl reagent according to the manufacturer’s instructions. Primer pair used for RT-PCR and sequencing: EBO6-SeqF1: 5ʹ TGTTCTCCCTGCCCATTAAG 3ʹ and EBO6-SeqR1: 5ʹ CTTGGAGATGGCATCAAAGG 3ʹ (for results see Supplementary Fig. 1c). The ratio of mutant-to-wild-type *Rps2* mRNA was quantified by RT-PCR (Supplementary Fig. 1d).

### Preparation of isolated mitochondria from skeletal muscle

Mitochondria were isolated from the muscle as previously described^59^. Briefly, muscle was removed from the quadriceps and gastrocnemius (~10–100 mg), and washed in 5 ml of ice-cold PBS/10 mM EDTA. Fat and collagen were removed by trimming the muscle in small pieces. Cleaned muscle was then transferred into an ice-cold solution of PBS/EDTA 10 mM/0.05% accutase, and incubated for 30 min. After centrifugation at 200*g* for 5 min at 4 °C, the pellet was resuspended in 1 ml of buffer for muscle mitochondria isolation (MMI = 210 mM mannitol, 70 mM sucrose, 10 mM HEPES, 1 mM EDTA, 0.45% fatty acid-free BSA, 0.5 mM DTT, 5× Complete Protease Inhibitor (Roche Diagnostics)) and homogenized with a glass homogenizer (10–15 strokes, 400 rpm). Homogenates were centrifuged at 1450*g* for 7 min at 4 °C to remove nuclei and tissue particles; centrifugation was repeated with the supernatant fraction for 3 min. The resulting supernatant fraction was centrifuged at 10,000*g* for 5 min at 4 °C to pellet mitochondria. The resulting pellet was re-suspended in 1 ml of MMI and centrifuged at 1450*g* for 3 min at 4 °C to remove debris. The mitochondria‐enriched supernatant was centrifuged at 10,000*g* for 5 min at 4 °C to obtain the mitochondrial fraction. This fraction was resuspended in 300 μl of PBS, followed by determination of protein content.

### Oxygen consumption and ATP measurements in isolated brain mitochondria

Rates of oxygen consumption were measured in isolated mitochondria using a Seahorse Bioscience XF24 Analyzer, following the manufacturer’s protocol and as previously described^60^. Briefly, mitochondria were diluted 1:10 in cold 1× MAS containing 10 mM succinate, 2 mM malate, and 10 mM pyruvate. In all, 50 μl of mitochondrial suspension (5 μg mitochondrial protein/well) was delivered to each well of a XF Cell Culture microplate and centrifuged at 2000*g* for 20 min at 4 °C to let mitochondria adhere to the wells. After centrifugation, 450 μl of pre-warmed (37 °C) 1× MAS plus substrates were added to each well and the plate was incubated 5 min at 37 °C in a CO_2_-free incubator prior to the experiment. The plate was placed in a XF24 Analyzer and oxygen consumption rates were assessed under different respiratory states as described^60^, except that oligomycin was added to a final concentration of 2.5 μg/ml.

ATP content was determined using Vialight plus kit (Lonza) following the manufacturer’s instructions and normalization per protein content.

### Determination of superoxide anion radicals

MitoSOX™ Red (Thermo Fisher) reagent is a fluorogenic dye specifically targeted to mitochondria in live cells. Oxidation of MitoSOX™ Red reagent by superoxide produces red fluorescence. Muscle homogenate samples were adjusted to 1 mg protein/ml in HBSS. First, 150 μl of a 5 μM MitoSOX™ reagent working solution (prepared according to the manufacturer’s protocol) were added to 250 μl sample, followed by incubation at 37 °C for 10 min, protected from light. Then, samples were centrifuged for 3 min at 500*g*. After discarding the supernatant, the pellets were washed three times with 250 μl HBSS (3 min at 500*g)*. Finally, the samples were transferred into a 96-well plate (final volume 100 μl per well) and fluorescence was detected using the Victor X5 multiplate reader at 510 nm (excitation) and 580 nm (emission). The intensity of fluorescence is proportional to superoxide anion radicals in mitochondria.

### Histopathological analyses

Hindlimb skeletal muscles of Rps2 A226Y mutant mice of 12 months of age were histologically analyzed in comparison to age-matched wild-type littermates (A226Y mutant *N* = 5; WT *N* = 5). To this end, frozen sections (4 μm) of M. soleus and M. gastrocnemius were subjected to standard histochemical stains to screen for pathological changes (H&E; to assess fiber atrophy, dystrophy-related alterations such as de-/regenerating and necrotizing fibers, fiber splitting, internalization of fiber nuclei, increase of endomysial connective tissue), fiber type distribution (glycolytic versus oxidative; ATPase stains at pH 4.2 and 9.4), mitochondrial respiratory dysfunction (COX), accumulation of glycogen (PAS) or neutral lipids (ORO), and dysregulated autophagic flux (acidic phosphatase). Comprehensive histopathological workup of hindlimb muscles M. soleus and M. gastrocnemius did not reveal any pathological changes.

### Blood analyses

Whole blood was transferred into EDTA-coated tubes (EDTA Microvette, Sarstedt) and analyzed for hemogram parameters; plasma samples were snap frozen and used for analysis of creatine kinase (UniCell DxC 800 Synchron, Beckman Coulter). Corticosterone levels in blood and muscle were determined by Metabolon (Metabolon Inc., Pittsburgh, USA).

### Western blot

Muscle tissue was lysed on ice in RIPA lysis buffer (150 mM NaCl, 1% Triton X-100, 0.5% sodium deoxycholate, 0.1% SDS, 50 mM Tris pH8.0) with Roche complete protease inhibitor (Sigma-Aldrich) and HALT^TM^ Phosphatase Inhibitor Cocktail (Thermo Scientific). Tissue was disrupted by grinding with a pestle while in the lysis buffer. A small amount of aluminum oxide powder, roughly equal in size to the tissue sample, was added to the lysis buffer to facilitate grinding. Lysates were then centrifuged (15,000 *g*, 10 min), supernatant aspirated, and normalized to protein concentration as measured using Micro BCA Protein Assay Kit (Thermo Scientific). The specific antibodies used were: Total OXPHOS Rodent WB Antibody Cocktail (Abcam, ab110413), Tnnc1 (Abcam, ab137130), Tnni2 (Abcam, ab184554), Serca2 (Abcam, ab91032), Serca1 (Abcam, ab105172), S6 (CST, #2217), phospho-S6 (Ser235/236) (CST, #2211), 4E-BP1 (CST, #9644), phospho-4E-BP1 (Thr37/46) (CST, #9459), FoxO3a (CST, #2497), phospho-FoxO3a (Ser253) (Abcam, ab47285), ubiquitin (linkage-specific K48) (Abcam, ab140601), GSK-3β (CST, #9315), phospho-GSK-3β (Ser9) (CST, #9336), Akt (CST, #9272), phospho-Akt (Thr308) (CST, #4056), phospho-Akt (Ser473) (CST, #4060), PGC1α (Thermo Scientific, #PA5-38021), Parkin (CST, #2132), anti-beta Tubulin (Abcam, ab6046), Anti-Rabbit IgG (HRP) (Abcam, ab205715), Mouse TrueBlot^®^ ULTRA: Anti-Mouse Ig (HRP) (Rockland, 18-8817-33). Antibodies were first tested on mouse muscle tissue from a separate cohort as a control for efficacy. A two-sided, unpaired Student’s *t*-test was used to determine difference between analyzed samples.

### HEK 293 cell culture

HEK 293 cells to be used as controls in western blots were grown at 37 °C in DMEM plus 10% FBS in 100 mm cell culture dishes (VWR). Cells were collected at 80–90% confluency using RIPA lysis buffer (150 mM NaCl, 1% Triton X-100, 0.5% sodium deoxycholate, 0.1% SDS, 50 mM Tris pH8.0) with Roche complete protease inhibitor (Sigma-Aldrich) and HALT^TM^ Phosphatase Inhibitor Cocktail (Thermo Scientific) and normalized to protein concentration measured using Micro BCA Protein Assay Kit (Thermo Scientific).

### Statistics and reproducibility

Several independent clones of WT and Rps2-A226Y mice were used for the experiments; the number of independent clones used in each experiment is specified in the corresponding figure legend. Statistical analysis was performed in R version 3.2.3 and Microsoft Excel. An unpaired Student’s *t*-test was used to determine significant difference between analyzed samples, unless otherwise specified in the figure legend.

### RNA sequencing and transcriptome analysis

RNA sequencing (RNAseq) was performed by GATC (Konstanz, Germany) according to the Illumina RNA sequencing protocol. For mutation-related changes, comparisons were made between a *Rps2* A226Y expressing mutant group and a Cre-recombinase expressing wild-type group. For the 9 months time point, 6/7 sequenced mice from each genotype were used for the analysis; for the 15 months time point, 8 wild-type mice and 10 mutant mice were sequenced and analyzed. Differential gene expression analysis between groups was performed using the R/bioconductor package edgeR^61^. To evaluate functional changes, differentially expressed genes (*p* < 0.05) were mapped to known biological ontologies based on the GO, KEGG, and Wiki projects using gene annotation tool Enrichr, and to process networks using MetaCore (GeneGo, Thomson Reuters).

For age-related changes, C57BL/6 wild-type 9 and 15 months groups were compared to C57BL/6 wild-type mice of 3 and 19 months from another study of ours^27^. The gene expression in these four groups was compared by BGA^62^ using the made4 package (bioconductor.org) in R. First, expression data from each study were batch corrected using ComBat from the sva package (version 3.32.1) in R. To remove noise and focus on those genes which change with age, the 7500 genes with the largest difference in expression between the 3 and 19 months groups were compared between all four groups. The first axis of the BGA was visualized to illustrate the separation of age groups along the aging axis.

Gene co-expression analysis was performed using the CEMiTool package (version 1.11.1) available at Bioconductor^32^. A *p*-value = 0.2 was applied for filtering genes with low variance, and minimum module size was set to 50 genes.

### Animals for behavioral analysis

Mice were housed at 21–22 °C under a 12/12 h light–dark cycle (lights on at 8 p.m.) in groups of 2–5, unless individual housing was required by experimental protocols. Mice were examined during the dark phase of the cycle under indirect dim light (ca. 12 lux) unless indicated otherwise. A cohort of 13 female mice (7 A226Y mutant, 6 litter-mate controls) were used to assess body weight and growth from 4 to 13 months of age. A second cohort of 18 female mice (10 A226Y mutant, 8 litter-mate controls) was tested: in a large open field, with a mechanical grip force meter, in the light–dark transition test, on the elevated O-maze, in a small open field with home box, in a water-maze place navigation task, on the rotarod, and finally in a cued and contextual fear conditioning task. At the onset of the test series the mice were 11.5 months old, by the end they had reached an age of 12.8 months. All procedures were approved by University of Zurich Animal Welfare Officers and the Cantonal Veterinary Office of Zurich (licenses 44/2015 and 41/2018).

### Grip test

The grip meter consisted of a mechanical force meter (max. force: 300 g) that was positioned horizontally 9 cm above a wooden plate and attached to a metallic T-bar of 5 cm width and 3 mm thickness. Mice were held by the tail and allowed to grasp the bar with both forepaws. They were then gently pulled away until they released the bar. Five consecutive measurements were obtained each during 2 subsequent sessions with an interval of approximately 1 h.

### Rotarod

The digitally controlled Ugo-Basile Mouse Rota-Rod apparatus (Cat. No. 47600, Ugo Basile, 21025 Comerio VA, Italy, www.ugobasile.com) permitted to test 5 mice concurrently. The diameter of the drum was 30 mm. Six dividers with 25 cm diameter confined five lanes, each 57 mm wide. During each trial, the drum was accelerating from 4 to 40 revolutions per minute (RPM) over the course of 5 min. The latency until a mouse fell off the drum was recorded using a trip switch below the animals. Each mouse was submitted to 5 trials with an inter-trial interval of at least 30 min.

### Water-maze place navigation task

The round pool had a diameter of 150 cm with 68 cm high walls. It was filled with water (24–26 °C, depth 15 cm) which was rendered opaque by addition of 1 l of milk (UHT whole milk 3.5% fat, Coop, Switzerland). The white quadratic goal platform (14 × 14 cm) was made of metallic wire mesh and painted white. It was hidden 0.5 cm below the water surface in the center of one of the 4 quadrants, approximately 30 cm from the sidewall. Salient extra-maze cues were placed on the walls of the testing room. Animals performed 30 training trials (max. duration 120 s), 6 per day with inter-trial intervals of 30–60 min and varying starting positions. During the first 18 trials, the hidden platform was held in the same position (acquisition stage) and then moved to the opposite quadrant for the remaining 12 trials (reversal stage). The first 60 s of the first reversal trial served as probe trial to test for spatial retention.

### Cued and contextual fear conditioning

Four mice were tested in parallel in a Noldus EthoVision-based fear conditioning system (Noldus Information Technology, Wageningen, the Netherlands, www.noldus.com). The conditioning chambers (175 mm deep × 180 mm wide × 280 mm high) were enclosed in ventilated and sound-attenuated cabinets and had a floor consisting of stainless steel rods permitting the application of current. The training session consisted of a 60 s pre-exposure to the training context immediately followed by three 60 s training trials consisting of a 2500 Hz 85 dB tone (CS) lasting 30 s, co-terminating with a 2 s 0.25 A foot shock (US), and followed by a 30 s interval. To assess memory consolidation 24 h after training, the mice were re-exposed to the training chamber for 120 s without activation of the CS or US (context test). Thereafter the floor of the conditioning chamber was covered with plastic, some bedding material, and pebbles. Right and rear walls were decorated with a chessboard pattern. The mice were pre-exposed to this modified context for 60 s which was immediately followed by a 60 s CS presentation (tone test).

### Large open field

The round arena had a diameter of 150 cm and 35 cm high sidewalls made of white polypropylene. Each subject was released near the wall and observed for 10 min on 2 subsequent days.

### Small openfield with home box

The small open-field arena measured 48 cm × 48 cm (wide × long) confined by 32.5 cm high sidewalls. The home box measured 12 cm × 8 cm × 4 cm. It was placed in the home cage of individually housed mice at least 24 h before testing. Each subject was observed during 30 min.

### Light–dark transition test

The apparatus consisted of a transparent chamber (20 × 30 cm, 20 cm high) exposed to direct bright room light and connected to an enclosed dark box (20 × 15 cm, 20 cm high) via a small opening. Subjects were released in the bright compartment and observed for 5 min.

### Elevated O-maze

A 5.5 cm wide annular runway with an outer diameter of 46 cm was positioned 40 cm above the floor. Two opposing 90° closed sectors were protected by inner and outer walls (height 16 cm). The remaining two open sectors had no walls. Transition zones were defined as locations from which the subject could poke into an open sector while keeping its body partially protected between walls. Animals were released near a closed sector and observed for 10 min.

### Video tracking

During open field and water-maze tests, on the elevated O-maze, and during the light–dark transition test, the mice were video-tracked using a Noldus EthoVision XT system (Noldus Information Technology, Wageningen, the Netherlands, www.noldus.com). The system recorded position, object area, and the status of defined event recorder keys on the keyboard. Raw data were then transferred to public domain software Wintrack 2.4 (www.dpwolfer.ch/wintrack) for further analysis.

### Statistical analysis of behavioral and body weight data

Statistical analyses and graphs were produced using R version 3.2.3, complemented with the packages ggplot2, psych, and moments. Data were analyzed using a general linear model with genotype (mutant, control) as between-subject factor. Within-subject factors were added as needed to explore the dependence of group effects on time, or trial. Significant interactions were explored by splitting the model. Significant effects of factors with >2 categorical levels were further explored using pairwise *t*-tests. Variables with strongly skewed distributions or strong correlations between variances and group means were subjected to Box-Cox transformation before statistical analysis as indicated. The significance threshold was set at 0.05. The false discovery rate (FDR) control procedure of Hochberg was applied to groups of conceptually related variables within single tests to correct significance thresholds for multiple comparisons. Similarly, FDR correction was applied during post hoc testing.

### Ethical animal research statement

All experiments performed on *Mus musculus* C57BL/6 complied with ethical regulations for animal testing and research, and were approved by the Veterinary Office of the Canton of Zurich (licenses 29/2012 and 44/2015).

### Reporting summary

Further information on research design is available in the Nature Research Reporting Summary linked to this article.

## Supplementary information

Supplementary information.

Descriptions of Additional Supplementary Files.

Supplementary Data 1.

Reporting summary.

## Data Availability

RNA-Seq data are available through NCBI’s Gene Expression Omnibus (GEO) database, GEO Series accession number GSE156283. Source data are provided with this paper as supplementary data. Datasets generated during and/or analyzed during the study are available from the corresponding author on reasonable request.

## Code availability

There was no original custom code used in this analysis.
