# Supplementary material for: Random errors in protein synthesis activate an age-dependent program of muscle atrophy in mice
Source: Commun Biol. 2021 Jun 8;4:703. doi: 10.1038/s42003-021-02204-z (PMC8187632; doi:10.1038/s42003-021-02204-z)
Supplement: Supplementary file 1 — Supplementary information. [file 42003_2021_2204_MOESM1_ESM.pdf]

Supplementary Table 1

Selected enrichment terms	p-value
Mitochondrion (GO:0005739)	1.28E-117
Oxidative phosphorylation_mmu00190 (KEGG)	1.01E-36
Mitochondrial translation (GO:0032543)	1.11E-32
Citrate cycle (TCA cycle)_mmu00020 (KEGG)	6.16E-17
Muscle contraction (GO:0006936)	9.49E-03
Pyruvate metabolism_mmu00620 (KEGG)	5.05E-07
Amino Acid metabolism_WP662 (Wiki)	2.57E-04
Fatty Acid Beta Oxidation_WP1269 (Wiki)	6.53E-03
Glycolysis / Gluconeogenesis_ mmu00020 (KEGG)	3.73E-02
Protein folding_Metacore Process Networks	2.133E-04

Supplementary Table 1: Curated gene enrichment list of upregulated terms comparing 9 months A226Y versus WT ($p<0.05$); FDR adjusted p-values are shown.

Supplementary Table 2

Selected upregulated enrichment terms	p-value
Ubiquitin-dependent protein catabolic process (GO:0006511)	2.43E-23
Proteasome_mmu03050 (KEGG)	7.25E-15
Autophagy_mmu04140 (KEGG)	1.22E-09
Mitophagy_mmu04137 (KEGG)	1.19E-08
Spliceosome_mmu03040 (KEGG)	1.01E-26
RNA transport_mmu03013 (KEGG)	1.54E-22
rRNA processing (GO:0006364)	1.16E-21
Cell cycle_Mitosis_Metacore Process Networks	5.32E-06
DNA damage_DBS repair_Metacor Process Networks	1.32E-04
Cell cycle_mmu04110 (KEGG)	1.70E-03
**Selected downregulated enrichment terms**	**p-value**
Glycine, serine and threonine metabolism_mmu00260 (KEGG)	1.38E-08
Arginine and proline metabolism_mmu00330 (KEGG)	5.06E-05
Tryptophan metabolism_mmu00380 (KEGG)	8.01E-05
Arginine biosynthesis_mmu00220 (KEGG)	0.005535

Supplementary Table 2: Curated gene enrichment list of regulated terms comparing 15 months A226Y versus WT ($p < 0.05$); FDR adjusted p-values are shown.

Supplementary Figure 1

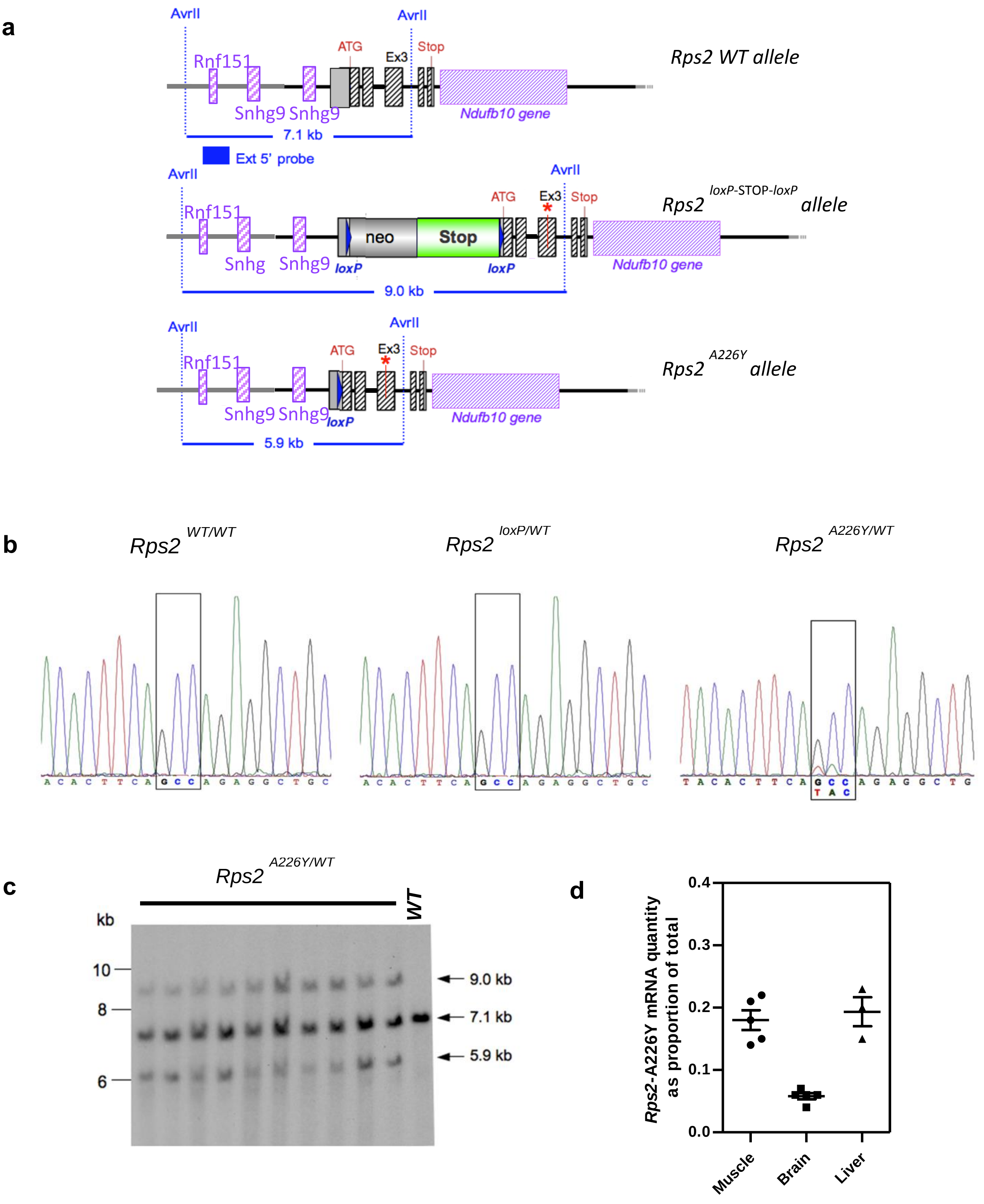

Supplementary Figure 1: Construction of the *Rps2*^{A226Y} mouse model (a) Schematic representation of WT, *Rps2*^{loxP} and *Rps2*^{A226Y} alleles. (b) Representative cDNA sequence analysis of WT, *Rps2*$^{flox/WT}$, and *Rps2*$^{A226Y/WT}$ mice. (c) Southern blot analysis of *Rps2*$^{A226Y/WT}$ mice genomic DNA compared to C57BL/6 wild-type DNA (WT). The *AvrII* digested DNA was blotted and hybridized with the external 5' probe. The 7.1 kb fragment indicates the wild-type allele, the 9.0 kb fragment the loxP allele and the 5.9 kb fragment the Rps2^{A226Y} allele. (d) Relative mRNA quantities (RT-PCR) of mutant *Rps2* A226Y mRNA compared to endogeneous wild-type *Rps2* mRNA in muscle, liver, and brain of heterozygous *Rps2*$^{A226Y/wt}$ mice. Mean ± SE ($3 \leq N \leq 5$).

Supplementary Figure 2

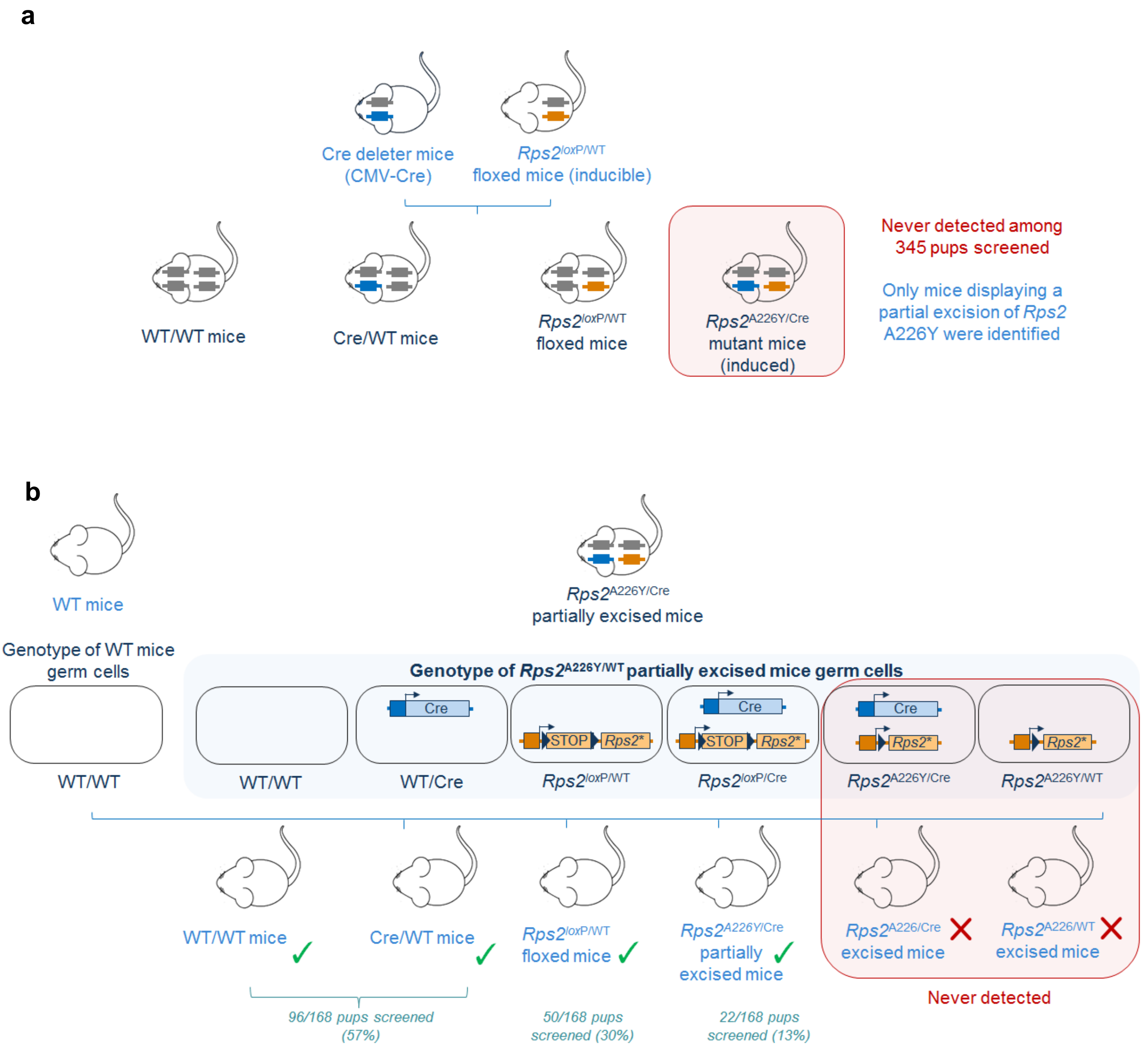

Supplementary Figure 2: Segregation breeding scheme (a) Breeding of heterozygous floxed *Rps2*$^{loxP/WT}$ mice with the CMV-Cre mice (b) Segregation breeding of partially excised *Rps2*$^{A226Y/WT}$ mice with wild type animals.

Supplementary Figure 3

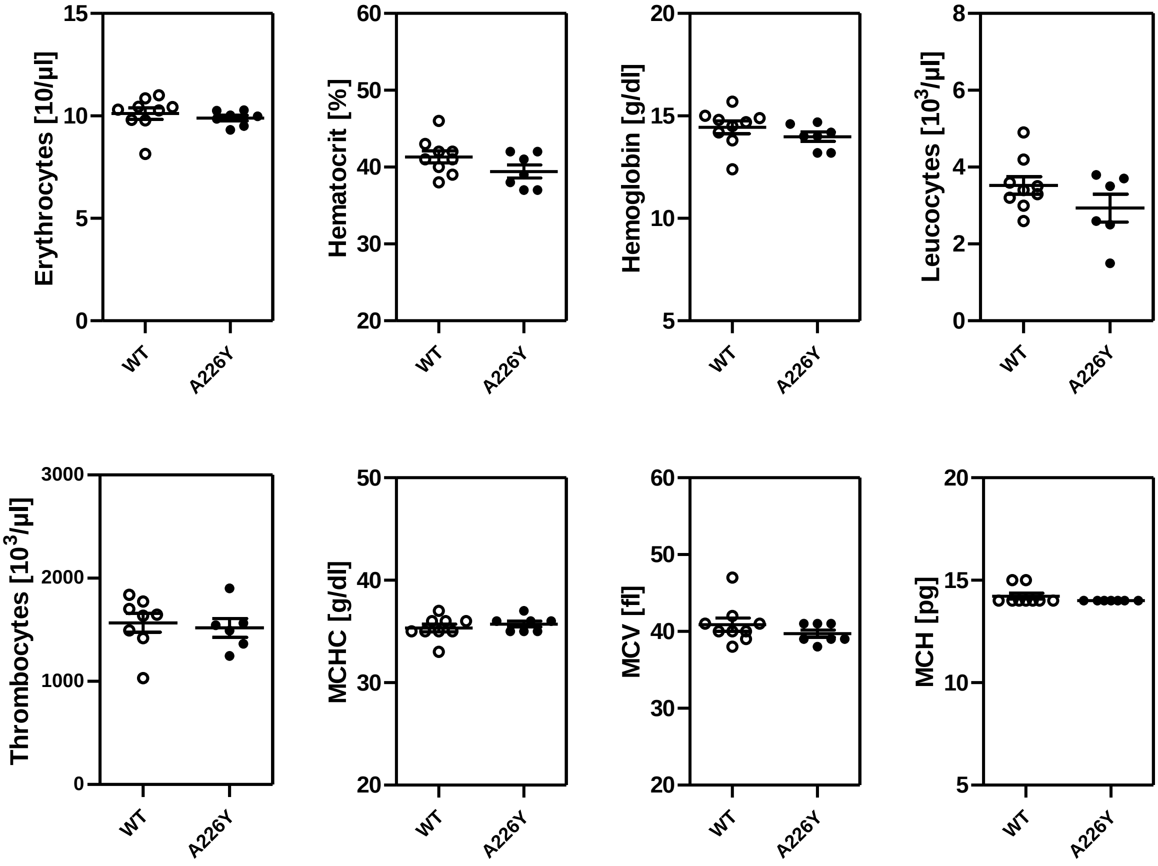

Supplementary Figure 3: Hematology measurements Graphs show mean, SE and individual data points. WT (*N*=9) and *Rps2* A226Y (*N*=7). No siginificant differences between A226Y and WT.

Supplementary Figure 4

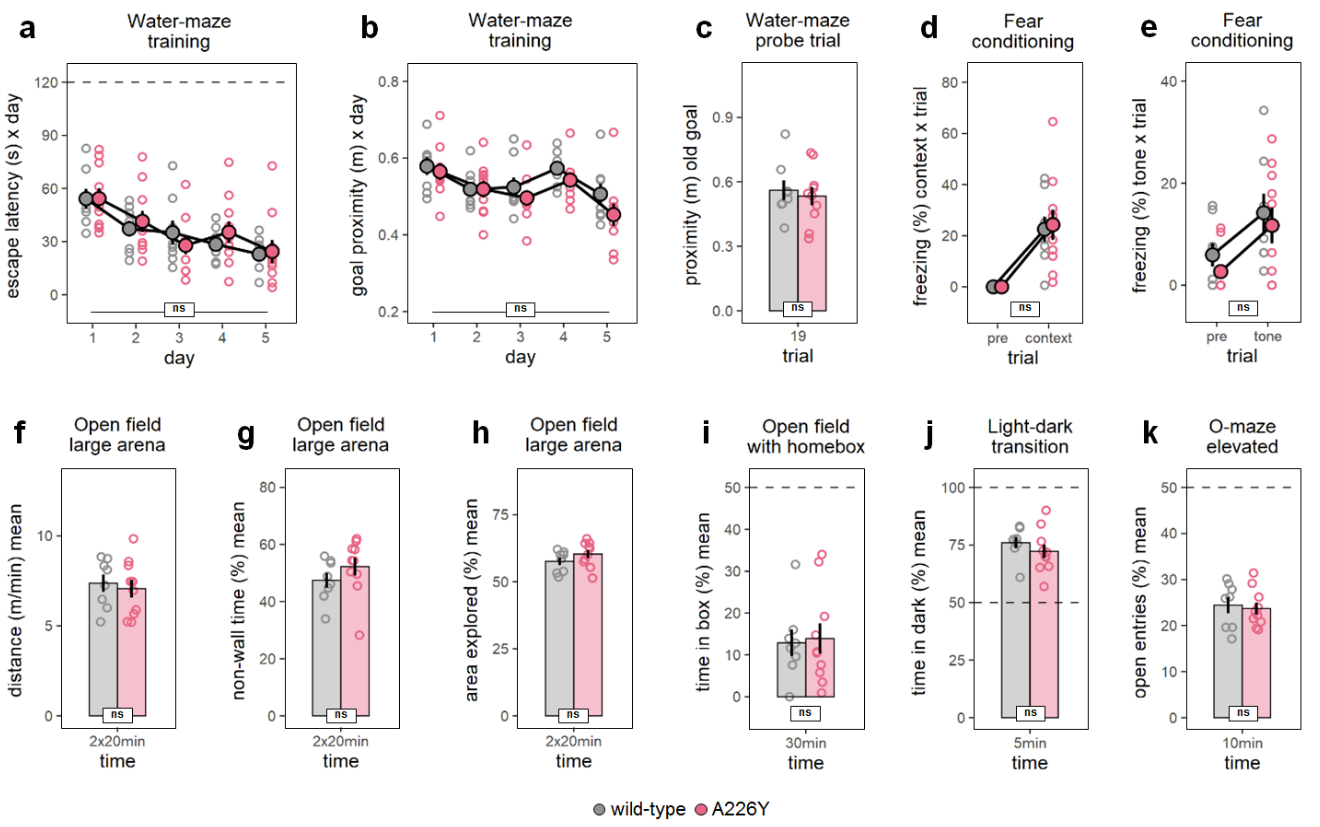

Supplementary Figure 4: Learning, memory, exploration and anxiety Graphs show untransformed mean, SE and individual data points (grey = wild-type mice, red = A226Y mice). ***p<0.001, **p<0.01, *p<0.05, ~p<0.1, ns p≥0.1. (a) Escape latency during acquisition (day 1-3) and reversal training (day 4-5) in the water-maze place navigation task. There was a significant decrease of escape latency during both training phases without evidence for a mutation effect on performance level or learning rate. (day $F_{4,64}=13.48$ $p<.0001$ $\eta^2=.46$, genotype $F_{1,16}=0.004$ ns, genotype x day $F_{4,64}=0.596$ ns, Box-Cox λ 0.5). (b) Goal proximity (average distance to goal) during acquisition and reversal training in the water-maze place navigation task. Proximity improved during acquisition and reversal learning, and worsened transiently on day 4 in response to goal relocation. There was no evidence for a mutation effect on level or improvement rate. (day $F_{4,64}=7.665$ $p<.0001$ $\eta^2=.32$, genotype $F_{1,16}=1.983$ ns, genotype x day $F_{4,64}=0.580$ ns, Box-Cox λ 0.0). (c) There was no evidence for a mutation effect on proximity to the previously trained goal during the water-maze probe trial, suggesting intact spatial retention (genotype $F_{1,16}=0.253$ ns, Box-Cox λ -2.0). (d) Freezing response to context (context re-exposure 24h post training versus pre-training baseline) during Pavlovian fear conditioning. There was no evidence for an effect of the mutation on the freezing response, indicating intact contextual memory (trial $F_{1,16}=35.33$ $p<.0001$ $\eta^2=.69$, genotype $F_{1,16}=0.050$ ns, genotype x trial $F_{1,16}=0.050$ ns). (e) Freezing response to tone (pre-tone versus tone re-exposure in novel context 24h post training) during Pavlovian fear conditioning. There was no evidence for an effect of the mutation on the freezing response, pointing to an intact conditioned response to the tone cue (trial $F_{1,16}=18.97$ $p<.0001$ $\eta^2=.54$, genotype $F_{1,16}=1.840$ ns, genotype x trial $F_{1,16}=0.013$ ns, Box-Cox λ 0.0). (f) Average distance moved per min observation time in a large open field arena (2x20 min on subsequent days) appeared unaffected by the mutation (genotype $F_{1,16}=0.191$ ns). There was also no evidence for a mutation effect on any of the assessed exploration and anxiety-related measures: (g) Time spent outside the 7cm wall zone of the large open field arena (genotype $F_{1,16}=1.297$ ns), (h) Average fraction of the large open field arena explored during 2x20 min of observation (genotype $F_{1,16}=1.705$ ns), (i) Fraction of time spent inside the home box during 30 min observation in the small open field (genotype $F_{1,16}=0.016$ ns, Box-Cox λ 0.0), (j) Fraction of time spent inside the dark compartment during the light-dark transition test (genotype $F_{1,16}=0.873$ ns), (k) Fraction of entries to open sectors during the elevated O-maze test (genotype $F_{1,16}=0.126$ ns).

Supplementary Figure 5

a

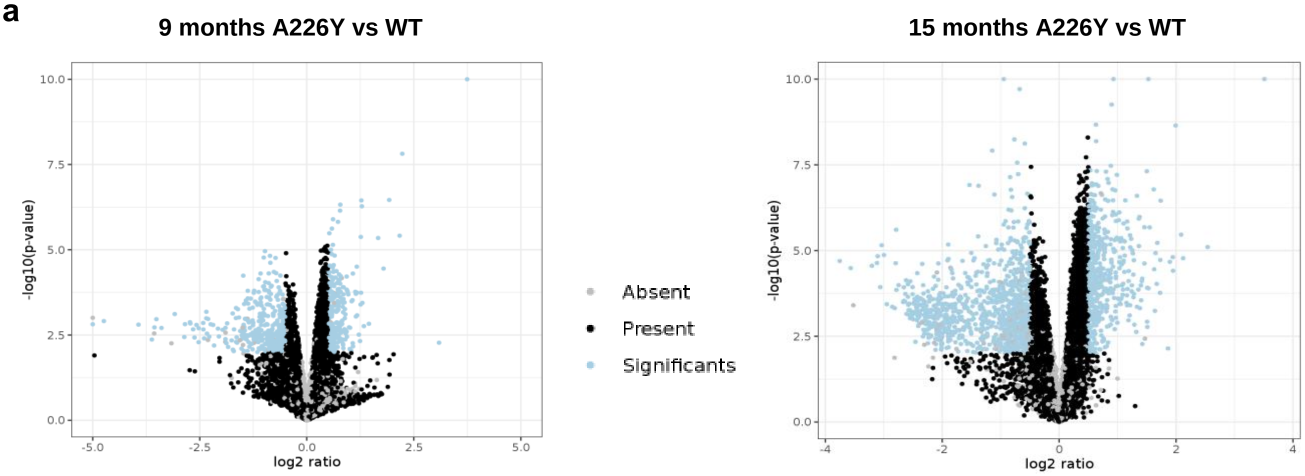

b

9 months A226Y vs WT

p-value	#sig	FDR	FC > 1.5	FC > 2
p < 0.05	3358	0.2078	729	234
p < 0.01	1589	0.0879	507	187
p < 0.001	467	0.0298	175	56

15 months A226Y vs WT

p-value	#sig	FDR	FC > 1.5	FC > 2
p < 0.05	6157	0.1148	1297	618
p < 0.01	4210	0.0336	1174	577
p < 0.001	2335	0.0061	800	386

c

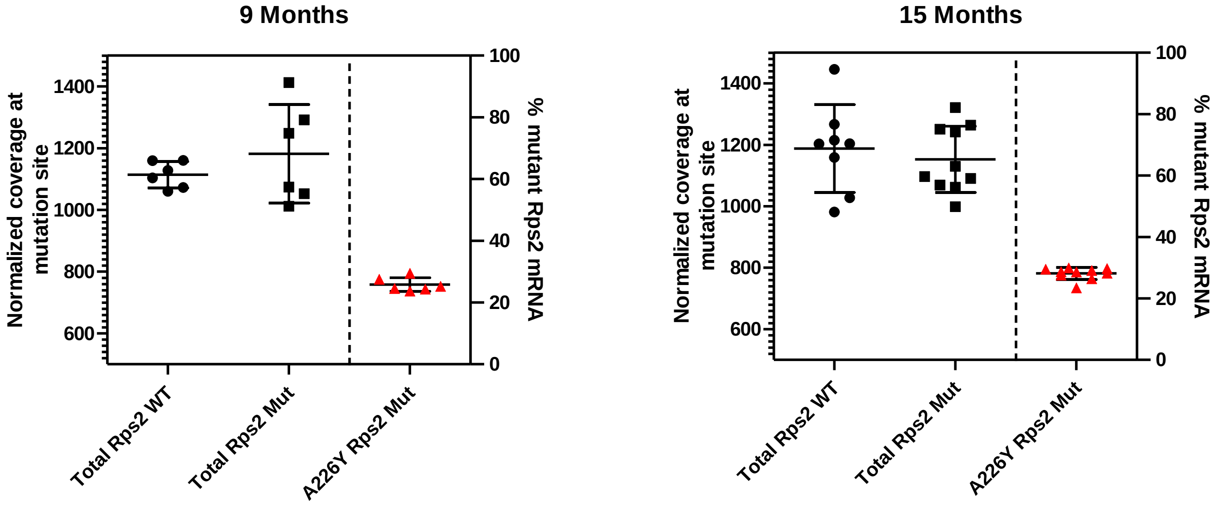

Supplementary Figure 5: Transcriptome overview of 9 and 15 months A226Y vs WT mouse muscle. (a) Volcano plot illustrating the differentially expressed genes. (b) Number of significantly differently expressed genes by p-value and fold-change. (c) Plots showing total Rps2 mRNA counts for each mouse from WT and mutant groups, and expression level of Rps2 A226Y in mutants as a percentage of total Rps2 (red); there is no difference in total Rps2 expression between mutant and WT groups. Plots show mean and SD.

Supplementary Figure 6

a

Downregulated with age in WT	p-value
mRNA processing (GO:0006397)	2.75E-17
mRNA splicing, via spliceosome (GO:0000398)	2.69E-16
Ribosome biogenesis (GO:0042254)	5.24E-09
RNA transport_mmu03013 (KEGG)	1.63E-05
Proteasome_mmu03050 (KEGG)	2.84E-14
Proteasome-mediated ubiquitin-dependent protein catabolic process (GO:0043161)	7.65E-12
Autophagy_mmu04140 (KEGG)	2.86E-04
Mitophagy_mmu04137 (KEGG)	5.21E-03
Oxidative phosphorylation_mmu00190 (KEGG)	8.87E-04
Regulation of cellular amino acid metabolic process (GO:0006521)	7.07E-13
Cell cycle_mmu04110 (KEGG)	8.32E-03

b

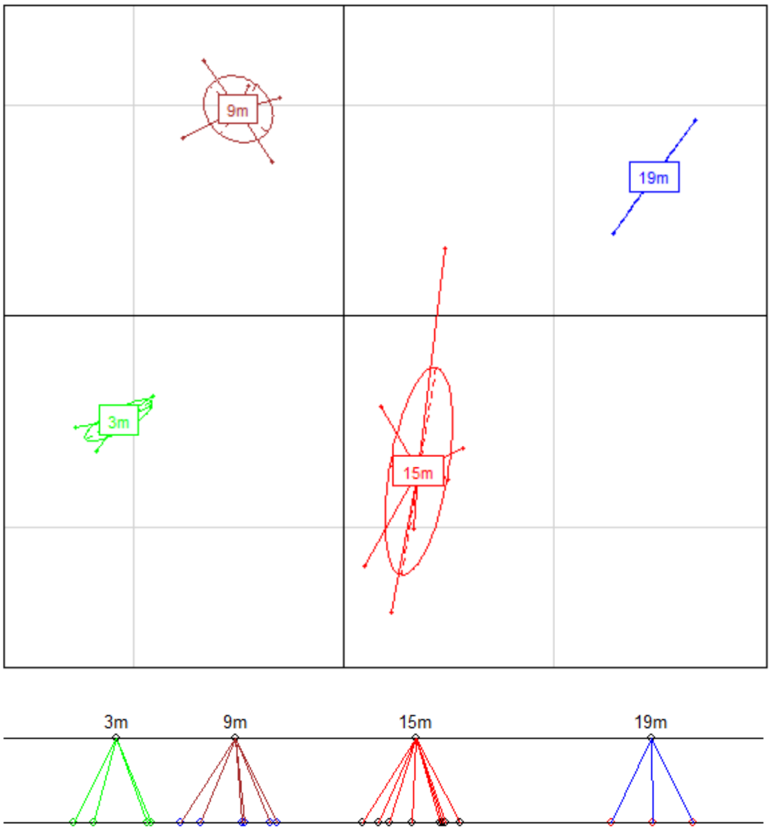

Supplementary Figure 6: Aging related transcriptomic changes in WT mice (a) Curated gene enrichment list of downregulated terms comparing 15 months WT versus 9 months WT ($p<0.05$); FDR adjusted p-values are shown. (b) BGA plot showing distribution of wild-type C57B/6 mice samples from the current study (9 and 15 months) compared to a previous study of 3 months and 19 months animals Shcherbakov et al. submitted for publication, number of genes = 7500. The horizontal axis corresponds to the age axis.

Supplementary Figure 7

a

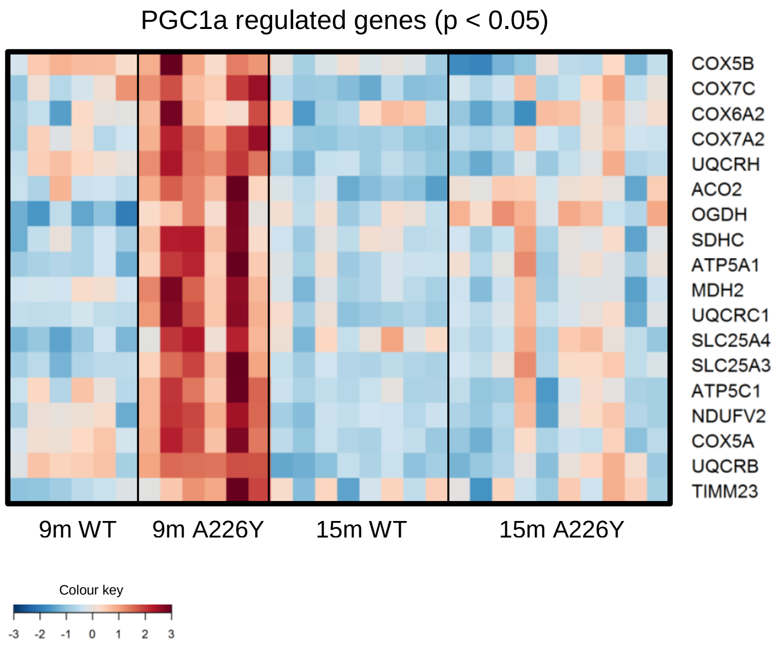

b

9 months A226Y vs WT

Gene	Adj. p-value	log2 Ratio
Cox5b	0.1987	0.2153
Cox7c	0.04675	0.3563
Cox6a2	0.08578	0.2932
Cox7a2	0.01298	0.5217
Uqcrh	0.02483	0.3132
Aco2	0.06555	0.3021
Ogdh	0.02787	0.3672
Sdhc	0.02234	0.4199
Atp5a1	0.02052	0.4666
Mdh2	0.03522	0.3388
Uqcrc1	0.01298	0.484
Slc25a4	0.02441	0.4669
Slc25a3	0.01307	0.5367
Atp5c1	0.05057	0.3036
Ndufv2	0.02165	0.4013
Cox5a	0.03522	0.3439
Uqcrb	0.02703	0.3118
Timm23	0.03634	0.4756

Supplementary Figure 7: PGC1a regulation in 9 and 15 months A226Y and WT mice (a) Heatmap representing genes regulated by PGC1a (*31*) including only genes with $p<0.05$ from 9 months A226Y vs WT (18/24). (b) Differential gene expression of PGC1a regulated genes in 9 months A226Y versus WT. FDR adjusted p-values and log2 ratio are shown.

Supplementary Figure 8

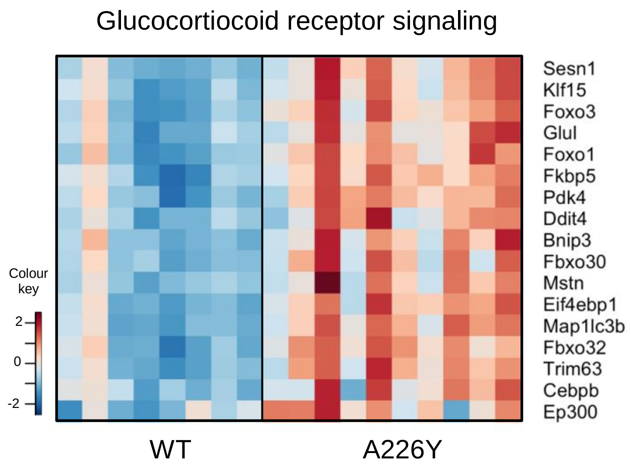

Gene	9 months A226Y vs WT adj. p-value	15 months A226Y vs WT adj. p-value
Sesn1	0.5091	0.000486
Klf15	0.08671	0.00115
Foxo3	0.6615	0.0005579
Glul	0.9176	0.003734
Foxo1	0.2562	0.0006362
Fkbp5	0.9795	0.001974
Pdk4	0.2297	0.001216
Ddit4	0.4905	0.006833
Bnip3	0.9809	0.002398
Fbxo30	0.8966	0.001681
Mstn	0.694	0.001293
Eif4ebp1	0.4883	0.0003738
Map1lc3b	0.9969	0.0002756
Fbxo32	0.6847	0.001425
Trim63	0.9356	0.001155
Cebpb	0.3978	0.00811
Ep300	0.3099	0.0101

Supplementary Figure 8: Heatmap of significantly upregulated (FDR < 0.05) glucocorticoid receptor signalling responsive genes, in 15 months WT and A226Y. Genes identified in the available literature (*1, 2, 3*). Table shows the differential expression between A226Y and WT groups for these genes, in both 9 months and 15 months mice. FDR adjusted p-values are shown.

Supplementary Figure 9

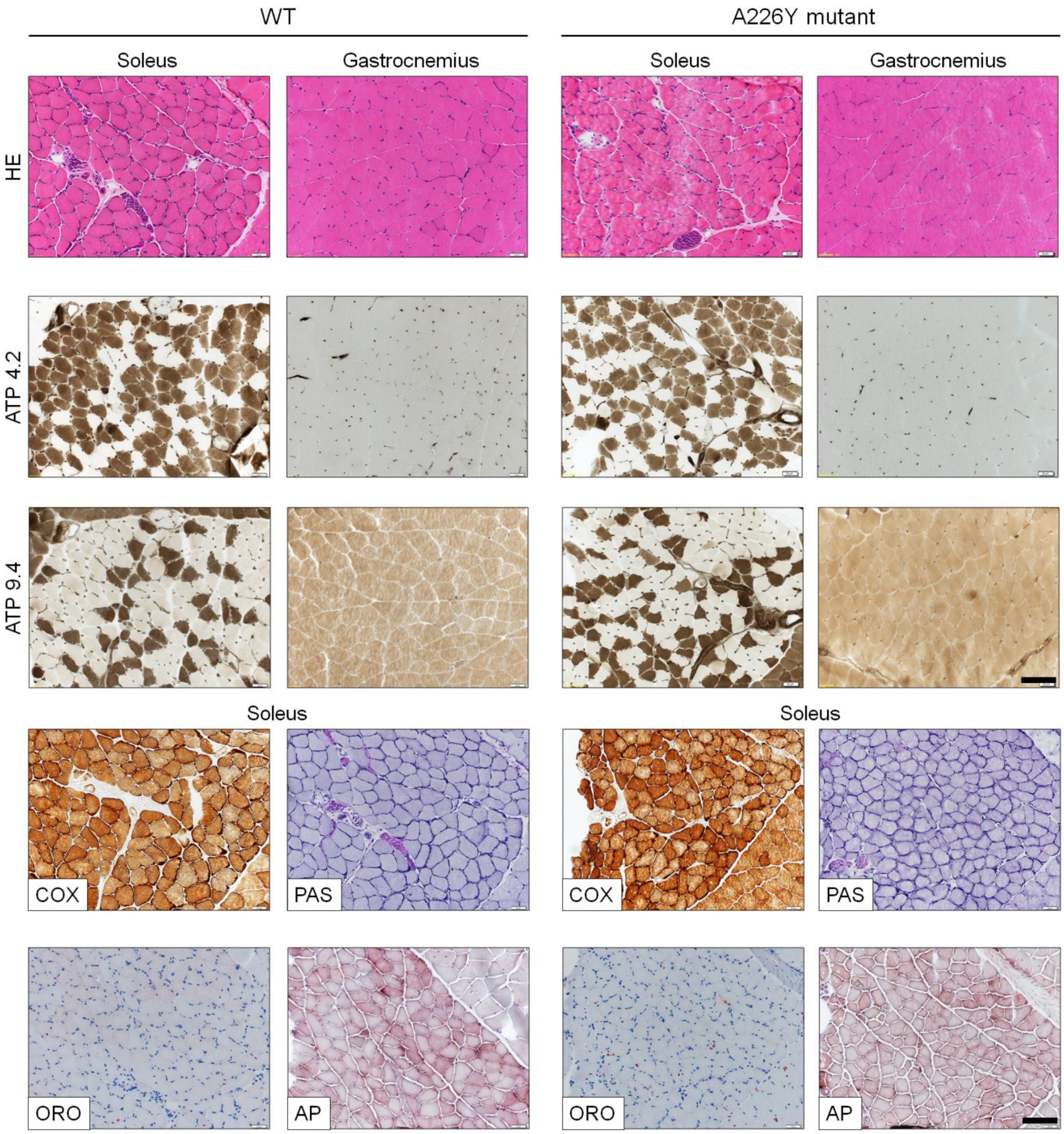

Supplementary Figure 9: Hindlimb muscle histology in age-matched wild-type (WT; column left) and A226Y mutant mice (right column). No abnormalities were noted on the morphological level (hematoxylin-eosin; H&E). Fibre type distribution (as judged by ATPase stains at pH 4.2 and 9.4), oxidative phosphorylation (assessed by COX-staining), and lysosomal activity (stains for acidic phosphatase; AP) were all normal. Likewise, no differences between genotypes were found in storage of glycogen (PAS) and neutral lipids (ORO). Scale bar, 50µm.

Supplementary Figure 10

a

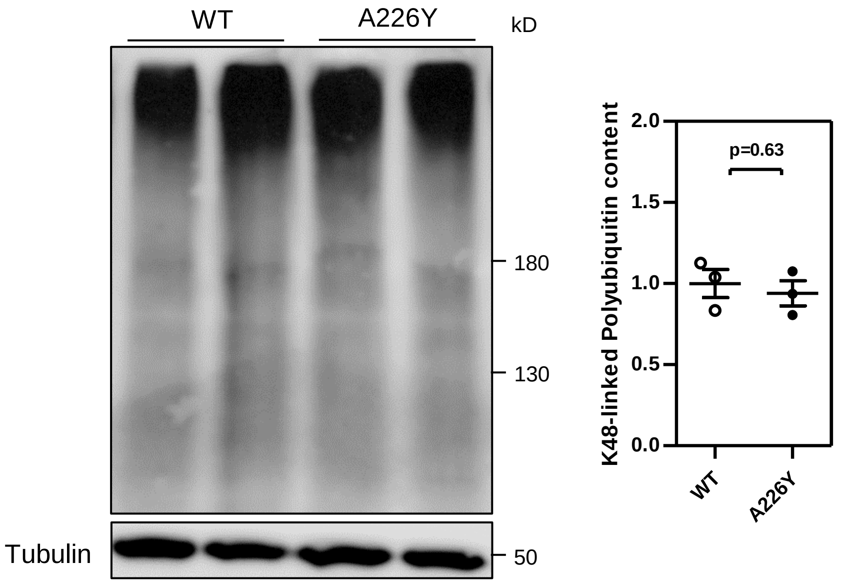

b

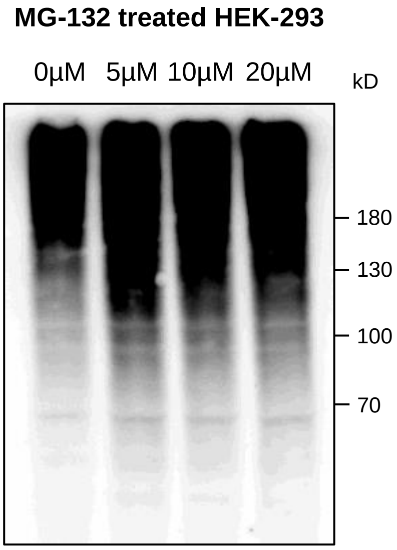

Supplementary Figure 10: Western blots of polyubiquitylated proteins Graphs show mean, SE and individual data points. (a) Western blot and densitometry of K48-linked polyubiquitin in 9 months WT and A226Y mice muscle tissue (*N*=3). Tubulin as loading control. (b) Western blot of K48-linked polyubiquitin in MG-132 treated HEK-293 cell culture (incubation with MG-132 at serial concentrations for 4 hours). MG-132 treatment was used as a positive control for the detection of K48-linked polyubiquitin.

Supplementary Figure 11

a

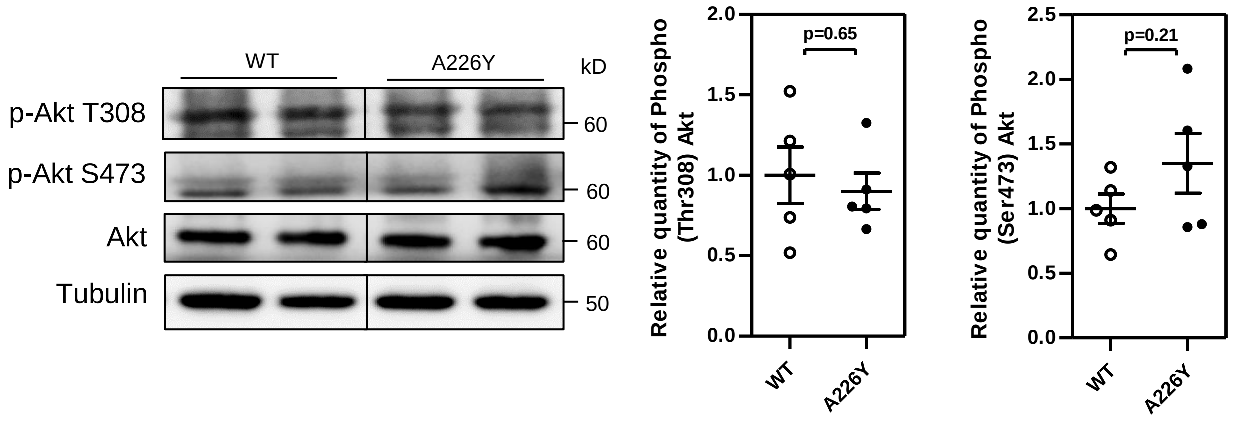

b

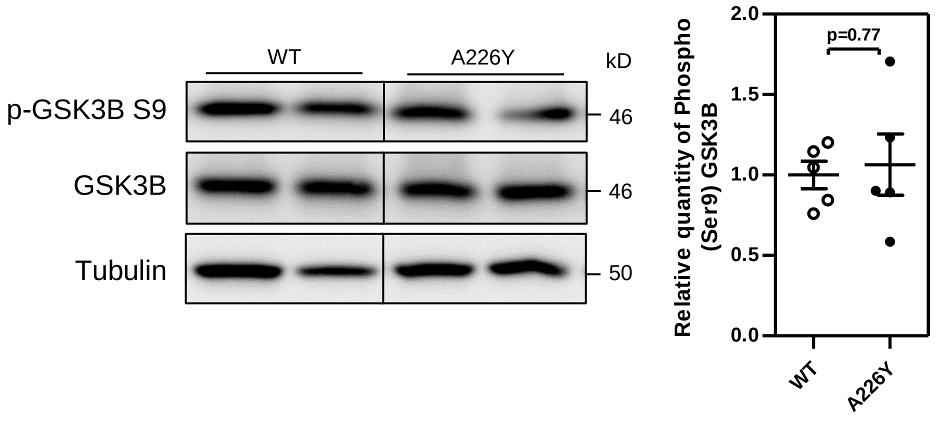

Supplementary Figure 11: Western blots showing no difference in the activation of key regulators Graphs show mean, SE and individual data points. Relative quantity of phosphorylated protein calculated as phosphorylated/total. (a) Western blots and densitometry of Akt and specific phosphorylated protein in 15 months WT and A226Y mice muscle tissue (*N*=5). Tubulin as loading control. (b) Western blots and densitometry of GSK3B and specific phosphorylated protein in 15 months WT and A226Y mice muscle tissue (*N*=5). Tubulin as loading control.

Supplementary Figure 12

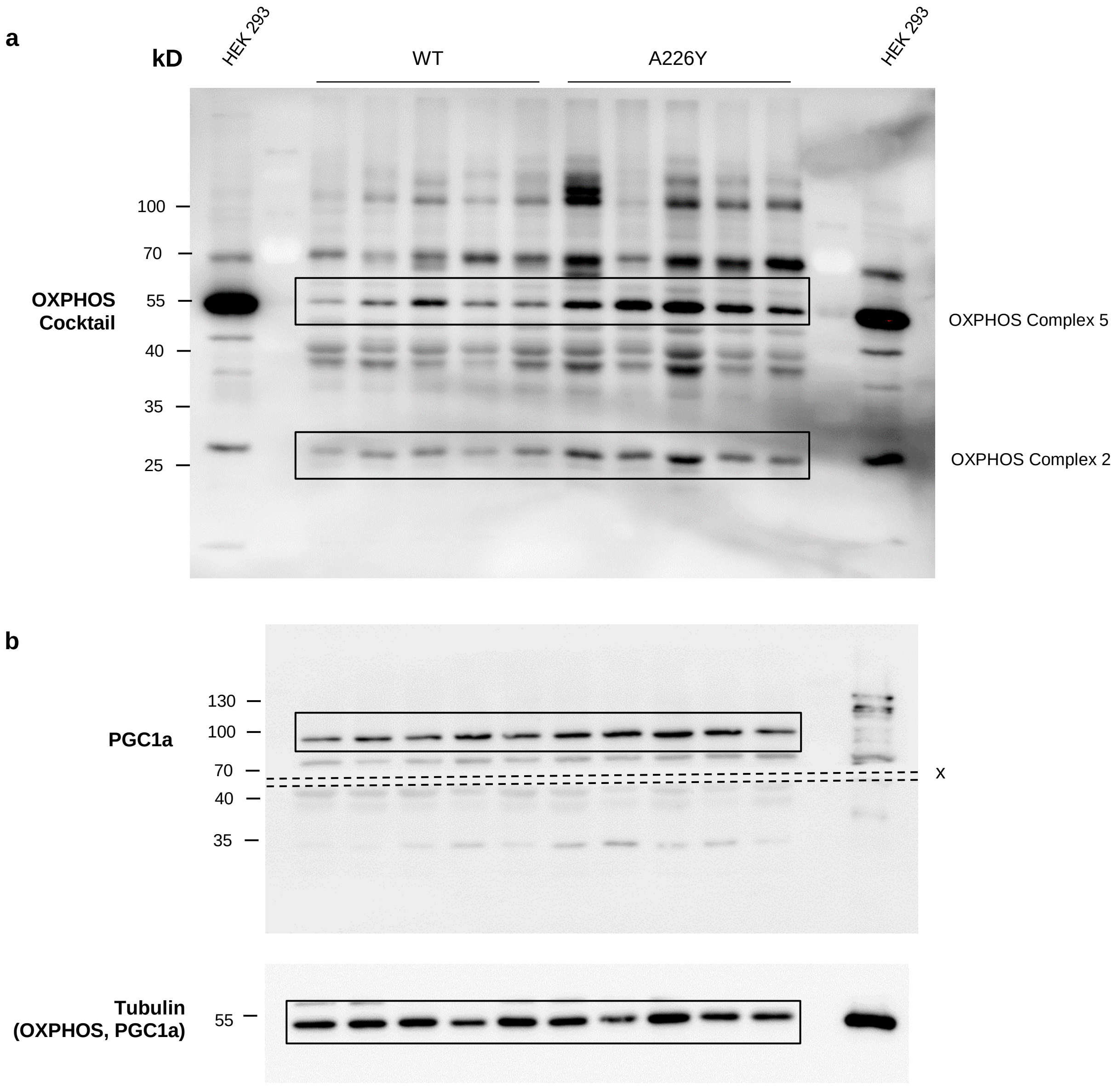

Supplementary Figure 12: Full gel images with corresponding molecular size markers from (a) Figure 2c (b) Figure 2f. Equal amounts of protein were loaded onto an SDS gel, run and transferred onto a NC membrane. HEK 293 cells were used as a control where indicated. Tubulin as loading control. ‘x’ marks where the membrane was cut to separate Tubulin for simultaneous detection.

Supplementary Figure 13

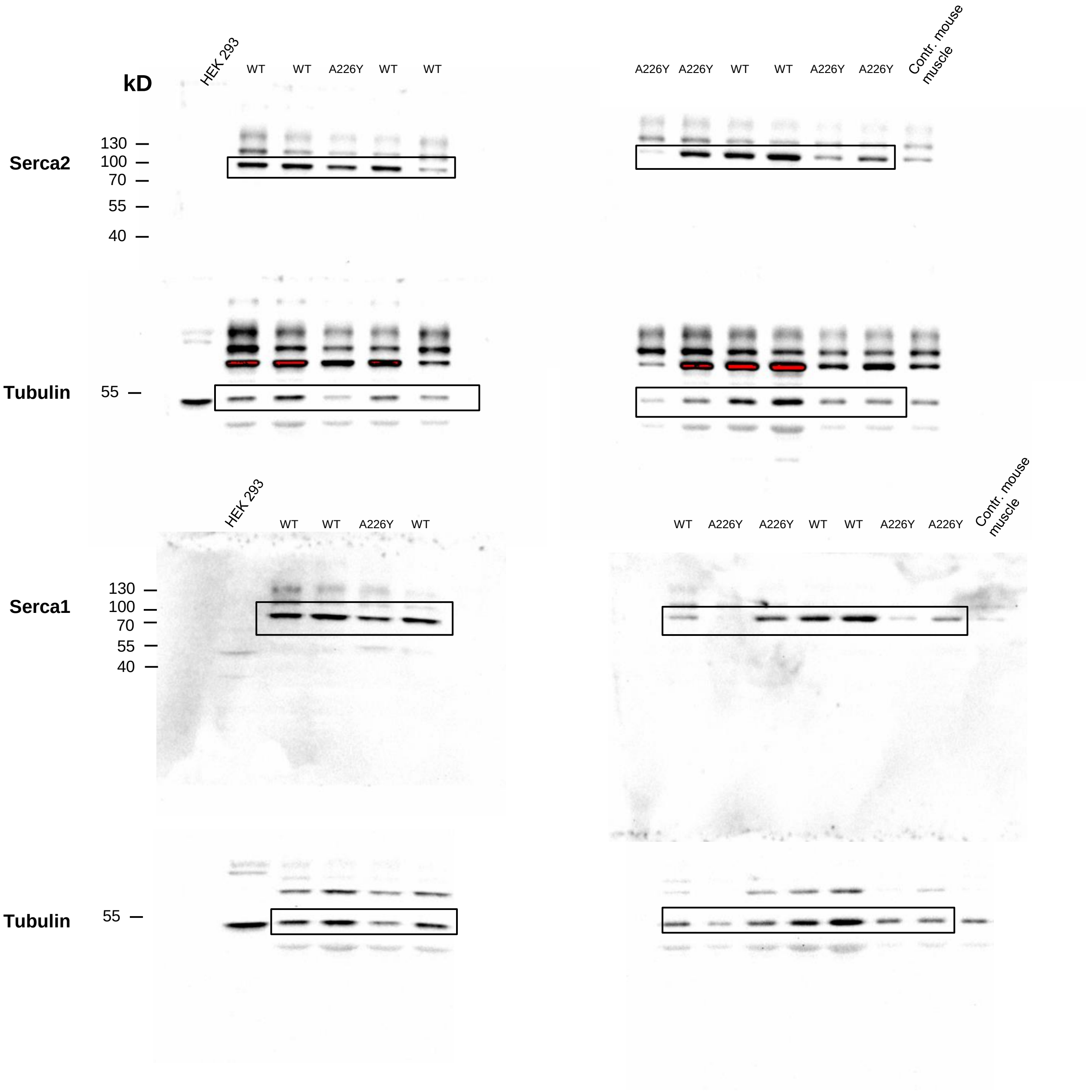

Supplementary Figure 13: Full gel images with corresponding molecular size markers from Figure 2e.

Protein was loaded onto an SDS gel, run and transferred onto a NC membrane. HEK 293 cells and control mouse muscle tissue were used as controls where indicated. Tubulin as loading control.

Supplementary Figure 14

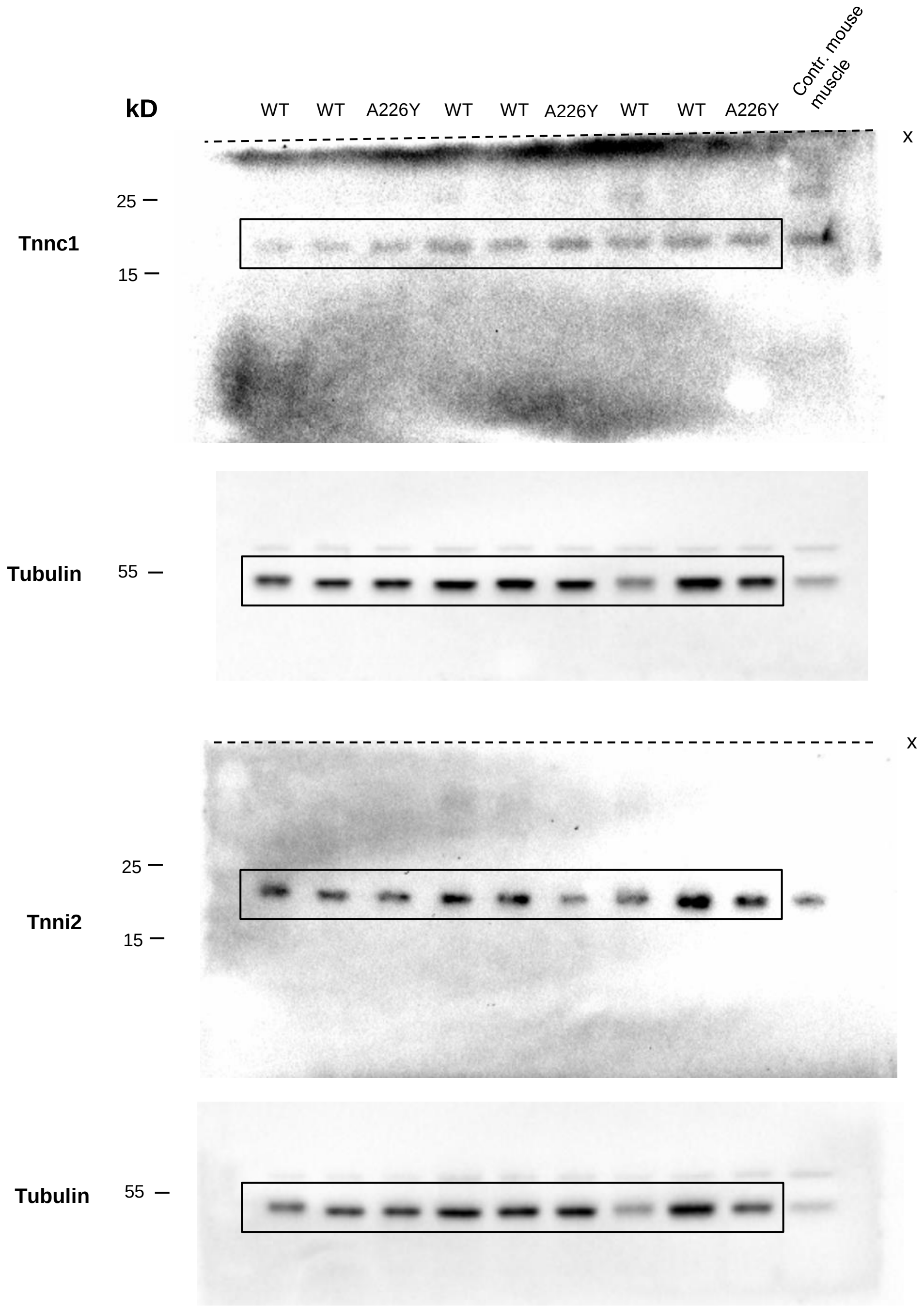

Supplementary Figure 14: Full gel images with corresponding molecular size markers from Figure 2e. Protein was loaded onto an SDS gel, run and transferred onto a NC membrane. Control mouse muscle tissue was used as a control where indicated. Tubulin as loading control. ‘x’ marks where the membrane was cut to separate Tubulin for simultaneous detection.

Supplementary Figure 15

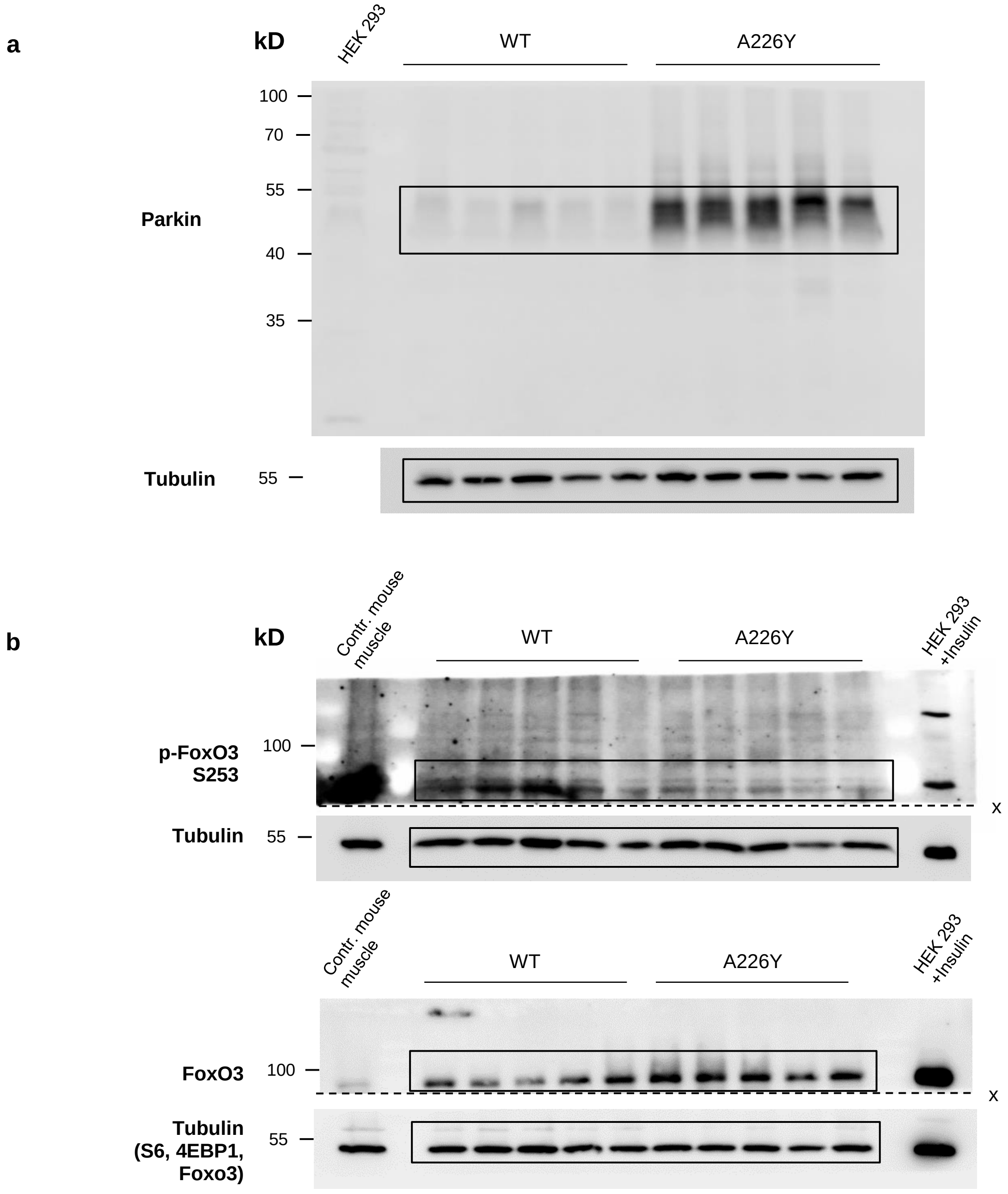

Supplementary Figure 15: Full gel images with corresponding molecular size markers from (a) Figure 3c (b) Figure 3e. Equal amounts of protein were loaded onto an SDS gel, run and transferred onto a NC membrane. HEK 293 cells, HEK 293 cells treated with Insulin (200nM, 30 minutes, following 12 hours serum starvation), and control mouse muscle tissue were used as controls where indicated. Tubulin as loading control. 'x' marks where the membrane was cut to separate Tubulin for simultaneous detection.

Supplementary Figure 16

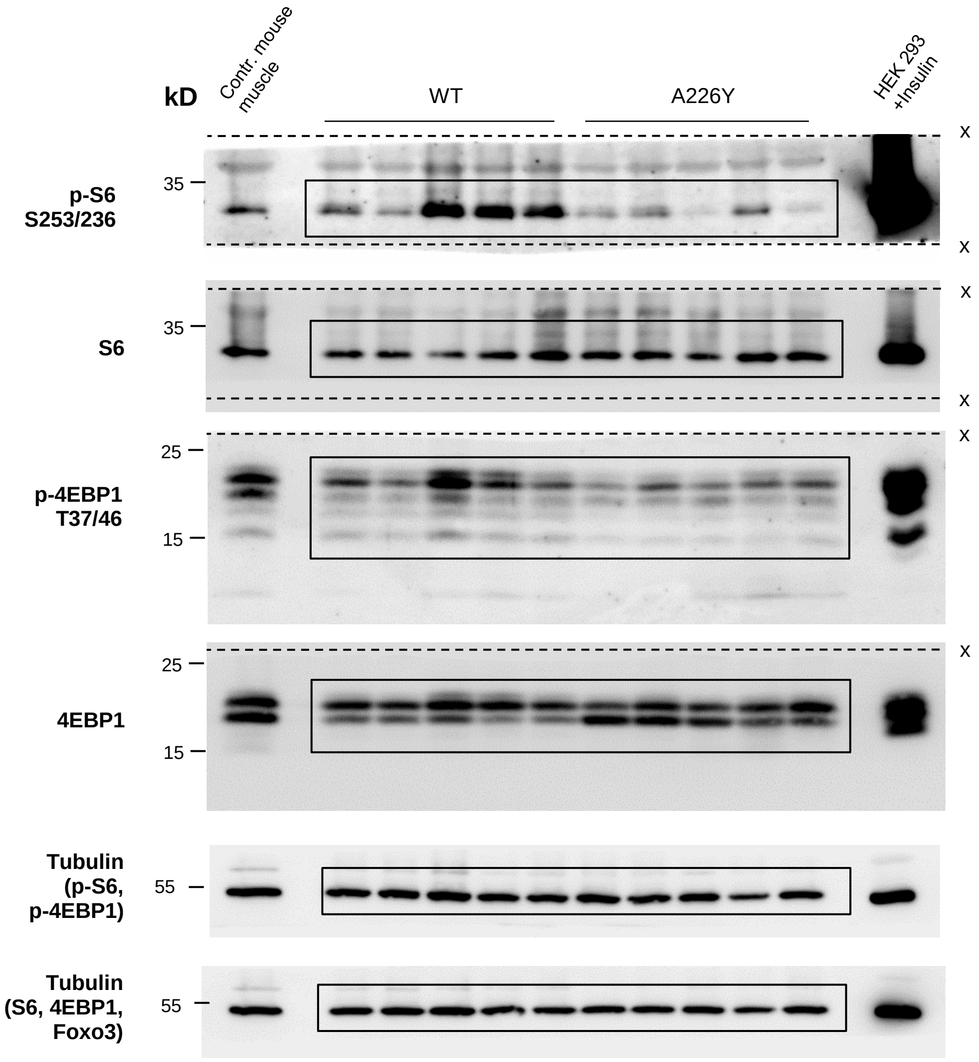

Supplementary Figure 16: Full gel images with corresponding molecular size markers from Figure 4b. Equal amounts of protein were loaded onto an SDS gel, run and transferred onto a NC membrane. HEK 293 cells treated with Insulin (200nM, 30 minutes, following 12 hours serum starvation) and control mouse muscle tissue were used as controls where indicated. Tubulin as loading control. ‘x’ marks where the membrane was cut to separate target proteins for simultaneous detection.

Supplementary Figure 17

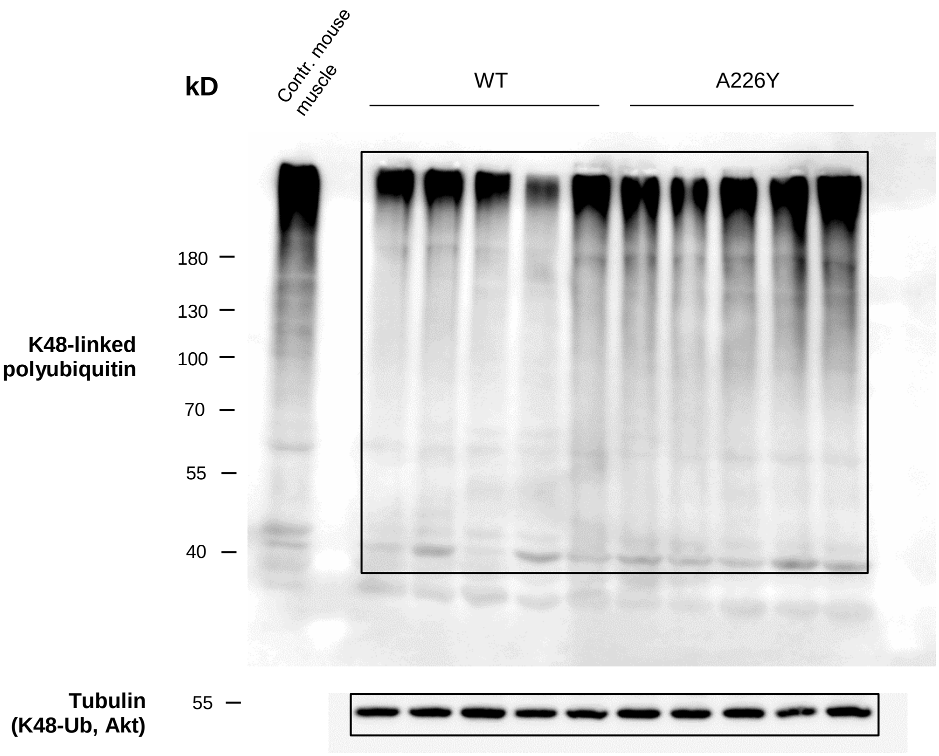

Supplementary Figure 17: Full gel images with corresponding molecular size markers from Figure 4c. Equal amounts of protein were loaded onto an SDS gel, run and transferred onto a PVDF membrane. Control mouse muscle tissue was used as a control where indicated. Tubulin as loading control.

Supplementary Figure 18

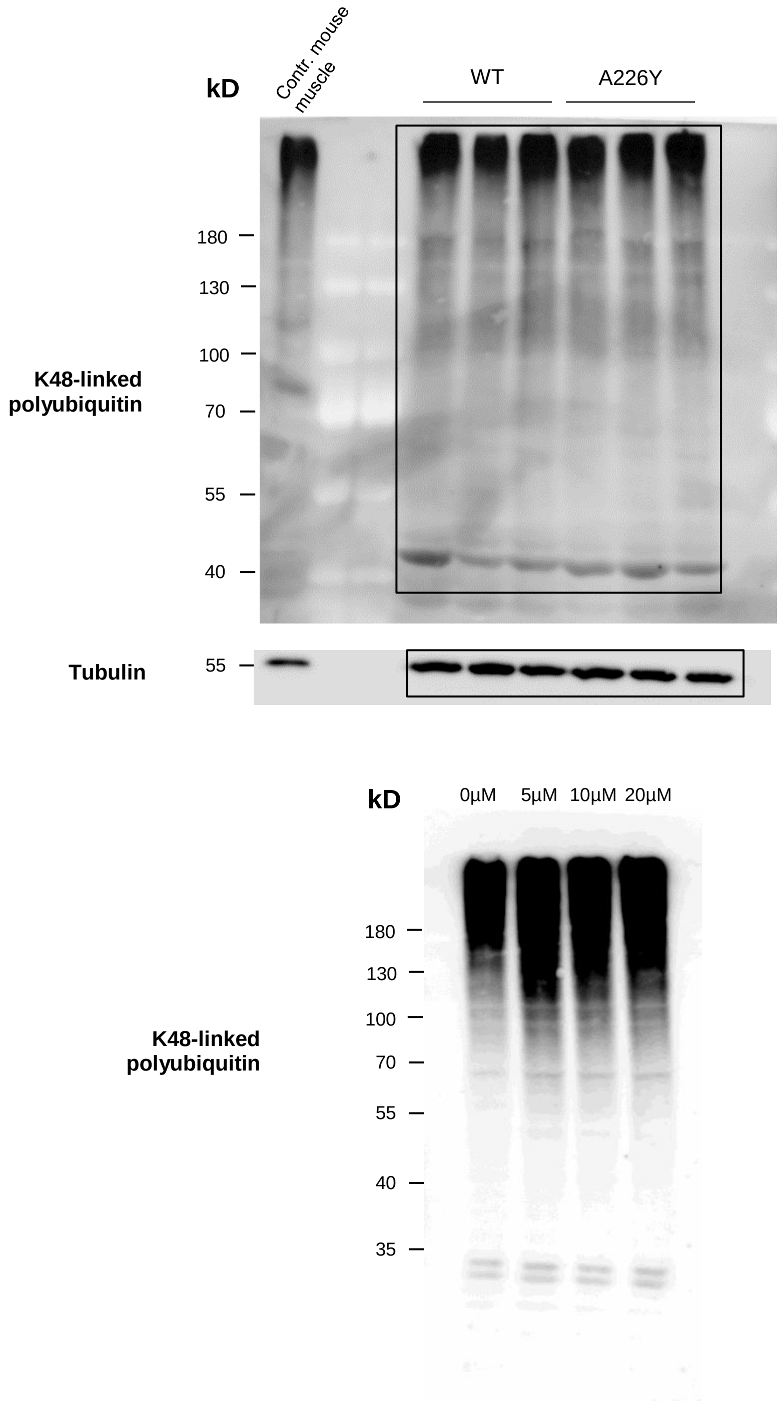

Supplementary Figure 18: Full gel images with corresponding molecular size markers from Supplementary Figure 10. Equal amounts of protein as measured by BCA were loaded onto an SDS gel, run and transferred onto a PVDF membrane. HEK 293 cells treated with MG-132 as a control for K48-linked polyubiquitin (concentration labelled, 4 hours). Control mouse muscle tissue was used as a control where indicated. Tubulin as loading control.

Supplementary Figure 19

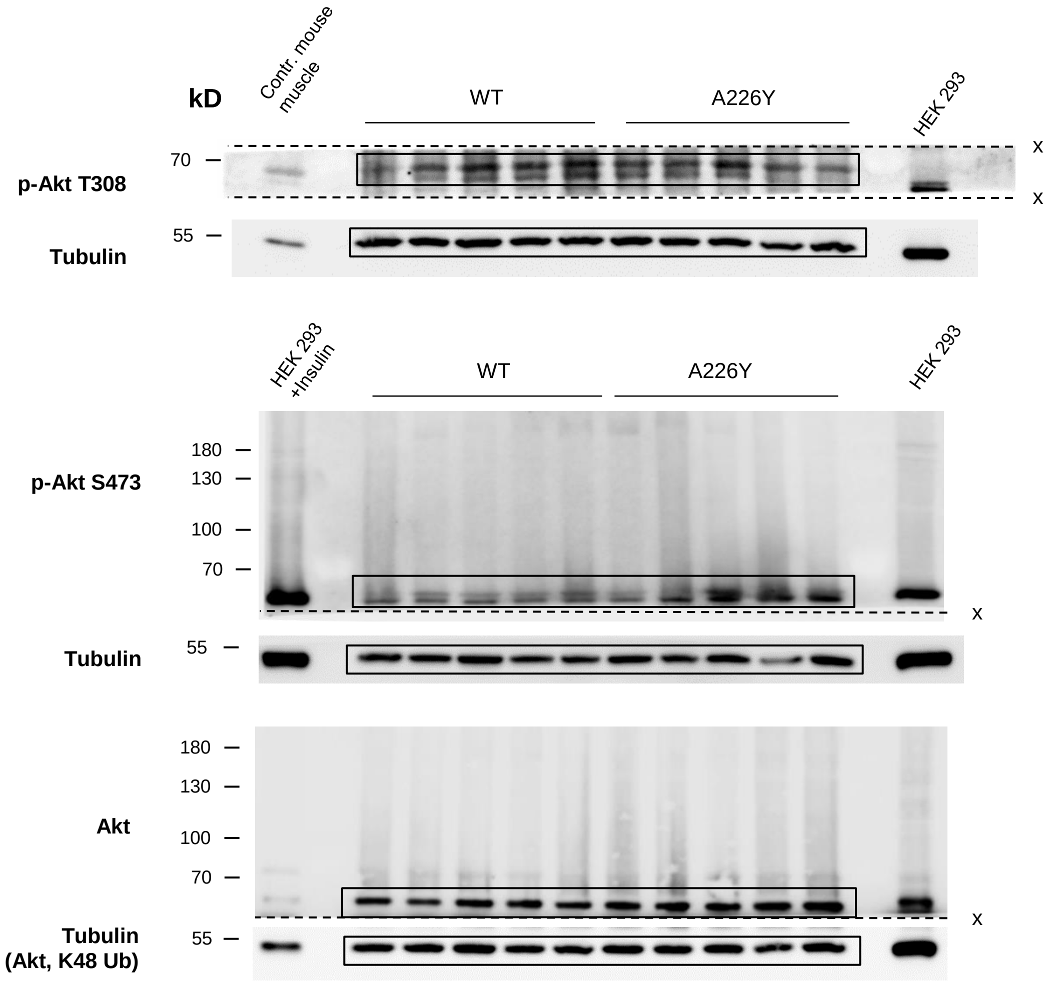

Supplementary Figure 19: Full gel images with corresponding molecular size markers from Supplementary Figure 11a. Full gel images with corresponding molecular size markers. Equal amounts of protein were loaded onto an SDS gel, run and transferred onto a NC membrane. HEK 293 cells, HEK 293 cells treated with Insulin (200nM, 30 minutes, following 12 hours serum starvation) or control mouse muscle tissue were used as controls where indicated. Tubulin as loading control. ‘x’ marks where the membrane was cut to separate proteins for simultaneous detection.

Supplementary Figure 20

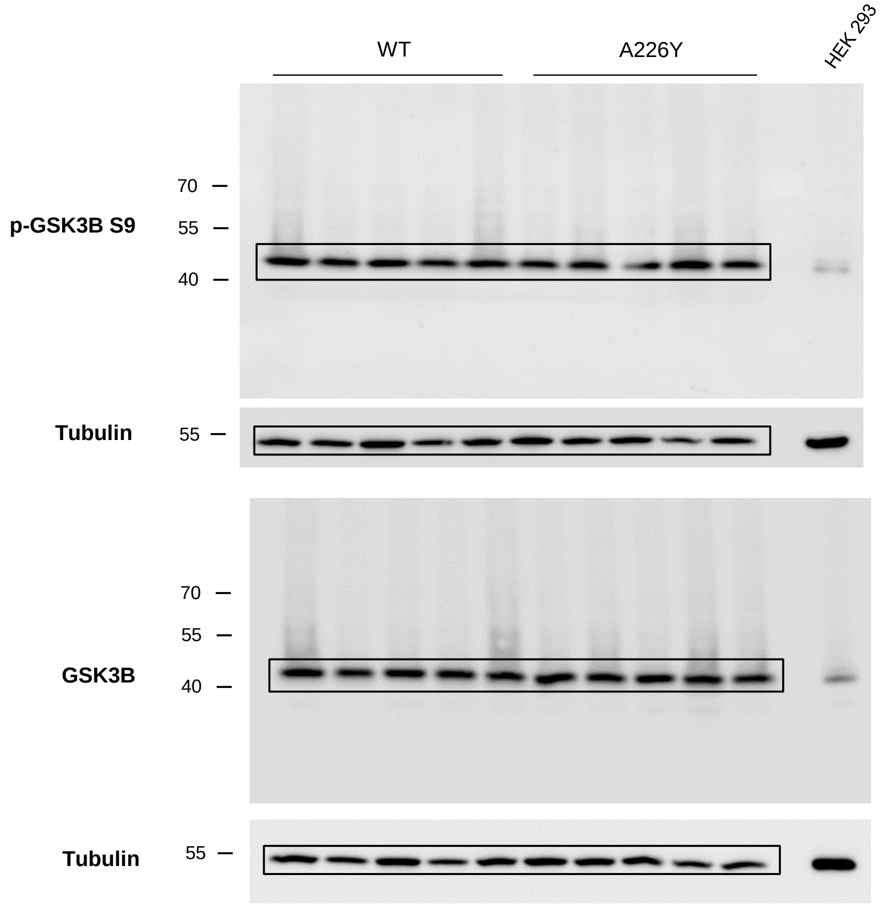

Supplementary Figure 20: Full gel images with corresponding molecular size markers from Supplementary Figure 11b. Equal amounts of protein were loaded onto an SDS gel, run and transferred onto a NC membrane. HEK 293 cells were used as a control where indicated. Tubulin as loading control.

References

1. Braun, T. P., & Marks, D. L., The regulation of muscle mass by endogenous glucocorticoids. *Front Physiol*. **3**, 12 (2015).
2. De Theije, C.C. *et al*. Glucocorticoid Receptor Signalling Impairs Protein Turnover Regulation in Hypoxia-Induced Muscle Atrophy in Male Mice. *Endocrinology* **159**, 519-534 (2018).
3. Braun, T. P. *et al*. Central nervous system inflammation induces muscle atrophy via activation of the hypothalamic-pituitary-adrenal axis. *J Exp Med* **208**, 2449-2463 (2011).
