# Supplementary material for: Random errors in protein synthesis activate an age-dependent program of muscle atrophy in mice
Source: Commun Biol. 2021 Jun 8;4:703. doi: 10.1038/s42003-021-02204-z (PMC8187632; doi:10.1038/s42003-021-02204-z)
Supplement: Supplementary file 2 — Descriptions of Additional Supplementary Files. [file 42003_2021_2204_MOESM2_ESM.pdf]

Description of Additional Supplementary Files

File name: Supplementary Data 1

Description: Source data underlying the main Figures.
