# Supplementary material for: Random errors in protein synthesis activate an age-dependent program of muscle atrophy in mice
Source: Commun Biol. 2021 Jun 8;4:703. doi: 10.1038/s42003-021-02204-z (PMC8187632; doi:10.1038/s42003-021-02204-z)
Supplement: Supplementary file 4 — Reporting summary. [file 42003_2021_2204_MOESM4_ESM.pdf]

nature research

Corresponding author(s): Prof. Dr. Erik C Böttger

Last updated by author(s): Apr 21, 2021

Reporting Summary

Nature Research wishes to improve the reproducibility of the work that we publish. This form provides structure for consistency and transparency in reporting. For further information on Nature Research policies, see our Editorial Policies and the Editorial Policy Checklist.

Statistics

For all statistical analyses, confirm that the following items are present in the figure legend, table legend, main text, or Methods section.

n/a	Confirmed	
☐	☒	The exact sample size (*n*) for each experimental group/condition, given as a discrete number and unit of measurement
☐	☒	A statement on whether measurements were taken from distinct samples or whether the same sample was measured repeatedly
☐	☒	The statistical test(s) used AND whether they are one- or two-sided<br>*Only common tests should be described solely by name; describe more complex techniques in the Methods section.*
☐	☒	A description of all covariates tested
☐	☒	A description of any assumptions or corrections, such as tests of normality and adjustment for multiple comparisons
☐	☒	A full description of the statistical parameters including central tendency (e.g. means) or other basic estimates (e.g. regression coefficient) AND variation (e.g. standard deviation) or associated estimates of uncertainty (e.g. confidence intervals)
☐	☒	For null hypothesis testing, the test statistic (e.g. *F*, *t*, *r*) with confidence intervals, effect sizes, degrees of freedom and *P* value noted<br>*Give P values as exact values whenever suitable.*
☒	☐	For Bayesian analysis, information on the choice of priors and Markov chain Monte Carlo settings
☒	☐	For hierarchical and complex designs, identification of the appropriate level for tests and full reporting of outcomes
☒	☐	Estimates of effect sizes (e.g. Cohen's *d*, Pearson's *r*), indicating how they were calculated

Our web collection on statistics for biologists contains articles on many of the points above.

Software and code

Policy information about availability of computer code

Data collection	Data was not collected using any code in this study.
Data analysis	There was no original custom code used in this analysis.<br>RNA-Seq read count normalization, quality control, and differential expression analysis were performed through the Functional Genomics Centre Zürich (FGCZ) user interface system Sushi, using FeatureCountsApp, CountQCApp and EdgeRapp respectively.<br>Heat maps and Between group analysis were done using the heatplot and bga function respectively in the R package made4, version 1.58.0.<br>The development version of CemiTool was used, available using devtools::install_github("csbl-usp/CEMiTool").

For manuscripts utilizing custom algorithms or software that are central to the research but not yet described in published literature, software must be made available to editors and reviewers. We strongly encourage code deposition in a community repository (e.g. GitHub). See the Nature Research guidelines for submitting code & software for further information.

Data

Policy information about availability of data

All manuscripts must include a data availability statement. This statement should provide the following information, where applicable:
- Accession codes, unique identifiers, or web links for publicly available datasets
- A list of figures that have associated raw data
- A description of any restrictions on data availability

RNA-Seq data will be available through the Gene Expression Omnibus (GEO) database, accession number GSE156283.

Field-specific reporting

Please select the one below that is the best fit for your research. If you are not sure, read the appropriate sections before making your selection.

- [x] Life sciences
- [ ] Behavioural & social sciences
- [ ] Ecological, evolutionary & environmental sciences

For a reference copy of the document with all sections, see nature.com/documents/nr-reporting-summary-flat.pdf

Life sciences study design

All studies must disclose on these points even when the disclosure is negative.

Sample size	The sample sizes used were the maximum number of mice we were capable of generating and analyzing according to the overall cost of the study and the facility capacities. This number of samples was sufficient for adequate statistical analysis.
Data exclusions	6/7 mice were used from A226Y and WT groups at the 9 month time-point. Mice were classified as outliers if they had a highly isolated pattern of gene expression, with low correlation to the rest of the group. This variability was deemed to be a technical artifact.
Replication	Findings were reproducible across the mice in our study, however due to obvious restrictions of time and money with generating another mouse cohort, we are unable to repeat the entire study.
Randomization	Randomization is not relevant to our study design for the comparison of a discrete group of mutant mice with a group of wild-type controls.
Blinding	Blinding was not necessary in this study as sufficient internal controls were in place to remove internal biases.

Reporting for specific materials, systems and methods

We require information from authors about some types of materials, experimental systems and methods used in many studies. Here, indicate whether each material, system or method listed is relevant to your study. If you are not sure if a list item applies to your research, read the appropriate section before selecting a response.

Materials & experimental systems

n/a	Involved in the study	
	x	Antibodies
	x	Eukaryotic cell lines
x		Palaeontology and archaeology
	x	Animals and other organisms
x		Human research participants
x		Clinical data
x		Dual use research of concern

Methods

n/a	Involved in the study	
x		ChIP-seq
x		Flow cytometry
x		MRI-based neuroimaging

Antibodies

Antibodies used	Total OXPHOS Rodent WB Antibody Cocktail (Abcam, ab110413), Tnnc1 (Abcam, ab137130), Tnni2 (Abcam, ab184554), Serca2 (Abcam, ab91032), Serca1 (Abcam, ab105172), S6 (CST, #2217), phospho-S6 (Ser235/236) (CST, #2211), 4E-BP1 (CST, #9644), phospho-4E-BP1 (Thr37/46) (CST, #9459), FoxO3a (CST, #2497), phospho-FoxO3a (Ser253) (Abcam, ab47285), ubiquitin (linkage-specific K48) (Abcam, ab140601), GSK-3B (CST, #9315), phospho-GSK-3B (Ser9) (CST, #9336), Akt (CST, #9272), phospho-Akt (Thr308) (CST, #4056), phospho-Akt (Ser473) (CST, #4060), PGC1a (Thermo Scientific, #PA5-38021), Parkin (CST, #2132), anti-beta Tubulin (Abcam, ab6046), Anti-Rabbit IgG (HRP) (Abcam, ab205715), Mouse TrueBlot® ULTRA: Anti-Mouse Ig (HRP) (Rockland, 18-8817-33).
Validation	All antibodies used have been validated by the supplier for use against mice in Western blotting.

Eukaryotic cell lines

Policy information about cell lines

Cell line source(s)	Parental cell line (HEK293) was purchased from ATCC.
Authentication	No authentication was required.
Mycoplasma contamination	The cell line used was tested for mycoplasma and found negative (mycoplasma-specific PCR and commercial antibody-based mycoplasma detection kit).
Commonly misidentified lines (See ICLAC register)	No commonly misidentified cell lines were used.

Animals and other organisms

Policy information about studies involving animals; ARRIVE guidelines recommended for reporting animal research

Laboratory animals	Mus Musculus, Female, C57BL/6, 9 months and 15 months of age.
Wild animals	The study did not involve wild animals.
Field-collected samples	The study did not involve samples collected from the field.
Ethics oversight	Animal experiments were approved by the Veterinary Office of the Canton of Zurich (licenses 29/2012 and 44/2015).

Note that full information on the approval of the study protocol must also be provided in the manuscript.
